# Pain alleviation in diabetic neuropathy using hybrid closed loop insulin pumps (PAINLESS) – A randomised crossover trial protocol

**DOI:** 10.1371/journal.pone.0357573

**Published:** 2026-09-08

**Authors:** Simon Alexander Berry, Ahmed Iqbal, Jackie Elliott, Rajiv Gandhi, Abdul Paracha, Vaios Koutroukas, Mohummad Shaan Goonoo, Mahnoor Fatima Haq, Andrew Gerges, Gordon Sloan, Solomon Tesfaye, Pratik Choudhary, Dinesh Selvarajah

**Affiliations:** 1 School of Medicine and Population Health, University of Sheffield, Sheffield, United Kingdom; 2 Department of Diabetes and Endocrinology, Sheffield Teaching Hospitals NHS Foundation Trust, Sheffield, United Kingdom; 3 Diabetes Research Centre, University of Leicester, Leicester, United Kingdom; National Healthcare Group, SINGAPORE

## Abstract

**Introduction:**

Painful diabetic neuropathy (PDN) is a common complication of diabetes that leads to significant co-morbidity. Emerging evidence suggests glycaemic variability may be linked to fluctuations in pain intensity. Hybrid closed loop (HCL) systems reduce glycaemic variability, raising the possibility that they may modify pain intensity. This study aims to evaluate the effects of HCL use on pain intensity in people with PDN.

**Materials and methods:**

PAINLESS is a two-centre, randomised cross-over study, recruiting 49 adults with type 1 diabetes and PDN. Each participant will complete two 12-week intervention periods in a randomised sequence (1:1): one with standard care including continuous glucose monitoring (CGM) and one with HCL using the Tandem t:slim X2 insulin pump with Control-IQ technology. The primary outcome is the change in 7-day average 24-hour pain intensity [rated on a numerical scale from 0-10 (NRS)] from baseline to the end of each 12-week intervention period as measured by daily pain diaries. Secondary outcomes include measures of pain trajectory/durability [responder rates (30% and 50% pain reduction in 7-day NRS), and the AUC for daily NRS scores], functional and quality of life outcomes [Short Form Health Survey (SF-36), and Short Form of the Brief Pain Inventory (BPI-MSF)], mood [Beck Depression Inventory (BDI)], health status [EQ-5D-5L], and global improvement [participant and clinician reported global impression of change]. Exploratory endpoints will harness neurophysiological assessments including Sudoscan, DPN-Check, Vagus and Vibrosense, and sleep and activity data from Withings devices. Statistical analyses will employ an intention-to-treat approach and mixed effect models.

**Timelines:**

24-month trial: 18 months recruitment/follow up, 6 months close-out, analyses and dissemination.

**Registration Details:**

ISRCTN24233750.

## Introduction

Diabetic peripheral neuropathy (DPN) is the most common complication of diabetes [1]. Although DPN can be asymptomatic, up to one-third have painful diabetic neuropathy (PDN) [2]. This is a disabling condition with a profound impact on quality of life, sleep, and mental health [3]. Current pharmacotherapy offers limited efficacy and tolerability [4] with only 54% of patients achieving 50% pain relief on maximal combination therapy [5]. Options for ‘refractory’ neuropathic pain (NeuP) are limited. Opioids carry significant risks and offer no long-term benefit [6], while spinal cord neuromodulation is invasive, costly and unsuitable for many [7]. Other interventions lack robust evidence or availability [4], leaving a major unmet clinical need.

Glycaemic variability (GV), reflecting fluctuations in glucose independent of overall glycaemic exposure, has been linked to fluctuations in pain intensity and has been identified as a potential modifiable therapeutic target [8]. Putative biological mechanisms to suggest the link between glycaemic variability and pathogenesis of diabetic neuropathy have been proposed, primarily via the induction of oxidative stress and systemic inflammatory responses [9]. Specifically, GV has been demonstrated to trigger the NF-kB and PKC pathways, leading to cellular damage that manifests as neuropathy. Continuous glucose monitoring (CGM) now allows precise measurement of GV, including coefficient variation (CV) and standard deviation of glucose (SD) [10]. A meta-analysis of 3,649 people with type 1 diabetes (pwT1D) and type 2 diabetes (T2D) demonstrated a two- to three-fold increased risk of DPN in those with higher GV [11]. Furthermore, GV has been specifically implicated in the development of painful over painless diabetic neuropathy [8,12]. Lower time in range (TIR), was significantly associated with an elevated risk and severity of PDN, independent of other risk factors [8]. However, whether reducing GV can directly modify pain intensity in PDN remains unknown.

Hybrid closed loop (HCL) insulin delivery systems automate basal insulin dosing using CGM data and predictive algorithms [13]. Randomised controlled trials consistently show that HCL improves overall glycaemic control and reduces GV [14–17]. This raises the possibility that HCL could modify pain intensity in PDN by stabilising glucose fluctuations.

The PAINLESS study is a two-centre, randomised cross-over trial designed to test this hypothesis. Adults with type 1 diabetes and PDN will receive 12-weeks of advanced HCL (Tandem t:slim X2 insulin pump with Control-IQ technology (Control-IQ)) and 12 weeks of standard care in randomised order. The primary outcome is the within-participant difference in 7-day average 24-hour pain on an 11-point numerical rating scale (NRS), consistent with IMMPACT recommendations for chronic pain trials [18]. Secondary outcomes include a core outcome set that will examine physical and emotional functioning and overall improvement. Finally, an embedded feasibility evaluation will assess acceptability, safety and operational considerations for future national health service roll-out.

## Materials and methods

### Trial design

**Fig 1:**
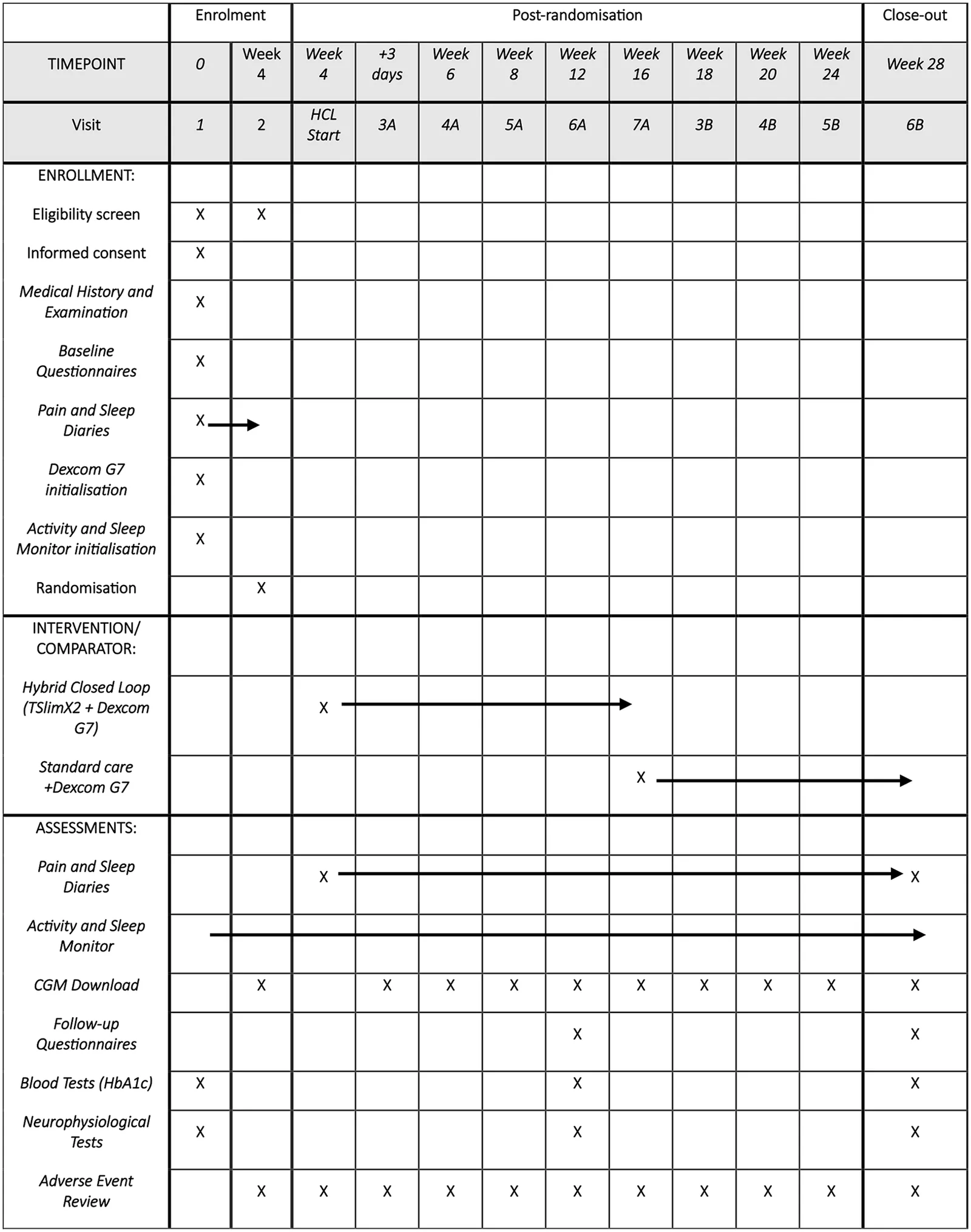
Participant timeline: Schedule of enrolment, interventions, and assessments in Spirit 2025 Format [19]. The study is a randomised crossover design. Only Schedule A is illustrated. In Schedule B, after randomisation participants start at visit 3B and complete the comparator arm first (up to visit 6B), before switching to HCL start and completing visits 3A-7A. In this case, visit 7A is the study close-out for the participant. HCL – Hybrid Closed Loop.

**Fig 2:**
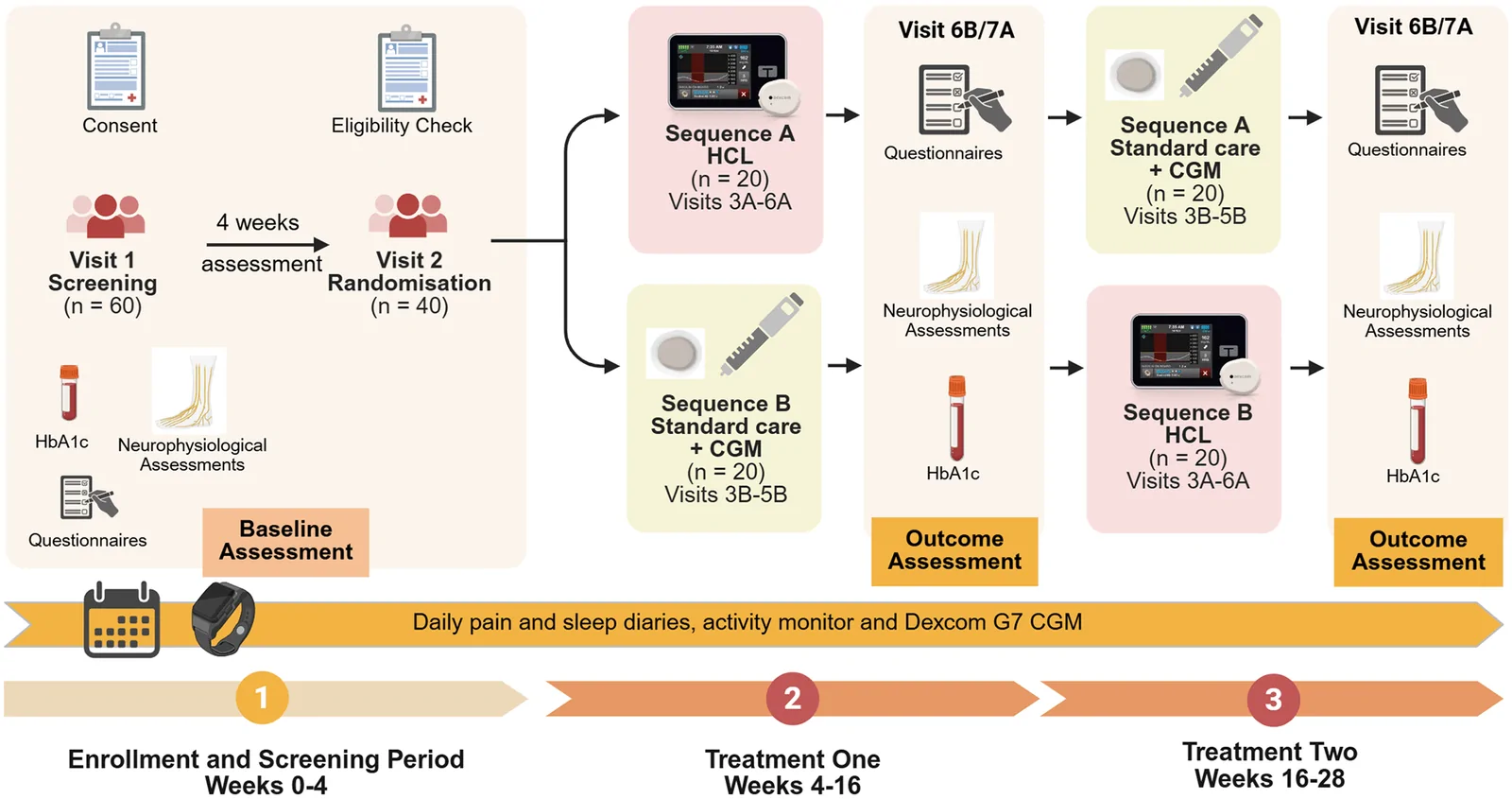
Flow Diagram of the PAINLESS Study. CGM – Continuous Glucose Monitoring, HCL Hybrid Closed Loop. Created in BioRender. Iqbal, A. (2026) https://BioRender.com/zh6dz7p.

Each participant will complete a four-week screening period prior to a 24-week treatment period (Figs 1, 2). During screening, participants will complete daily sleep and pain diaries and wear CGM and an activity tracker. Participants with an average pain NRS ≥ 4 in the last 7 days, will be randomised (1:1 ratio) to begin either standard care with CGM or HCL insulin treatment for the first 12 weeks, followed by the alternative intervention for the second 12 weeks. No washout period is planned between intervention arms.

### Eligibility criteria

The PAINLESS study will recruit adults with type 1 diabetes who have experienced daily neuropathic pain for at least 3 months (Table 1). People with type 2 diabetes are not included as the pathophysiology of PDN differs, with greater influence of metabolic syndrome and dyslipidaemia [20]. Eligibility criteria were designed to exclude confounding conditions and mitigate potential barriers to HCL use. Co-enrolment of participants in observational studies is permitted but co-enrolment in other non-pain studies would require trial management group (TMG) approval.

**Table 1:** Inclusion and exclusion criteria.

Inclusion criteria
• Age > 18 years.
• Clinical diagnosis of type 1 diabetes for at least 5 years [commenced on insulin treatment within one year of diagnosis].
• Probable neuropathic pain > 3 months confirmed by IASP (NeupSIG) criteria.
• Distal symmetrical polyneuropathy confirmed by the Toronto Clinical Neuropathy Score >5.
• Douleur Neuropathique 4 score >4 at screening visit to confirm NeuP diagnosis.
• HbA1c between 6.5 to 13% (47–118 mmol/mol).
• No change to dose of neuropathic pain medication within the last month.
• 7-day average pain NRS ≥ 4/10 during the final week of the Screening Period.
• Willing and able to comply with the study schedule and be available for the treatment duration.
• Able to give written informed consent.
**Exclusion criteria**
• Non-diabetic neuropathies.
• Predominantly nociceptive, mixed or other more severe pain (unless transient) – to ensure diagnostic specificity, and avoid confounding, as mechanisms and response differ from NeuP.
• Cognitive impairment or consent issues: to ensure ethical consent and valid, reliable self-report pain data.
• Pregnancy/breast feeding or planning pregnancy during the course of the study.
• Visual or hearing impairment, which would impede ability to use technological interventions

IASP – International Association for the Study of Pain; NeupSig – Neuropathic Pain Special Interest Group; NeuP – Neuropathic Pain; NRS – Numerical Rating Scale.

### Study setting and recruitment

To account for anticipated dropout and screen failure rate, the PAINLESS study will enrol approximately 49 participants in the screening period to achieve a randomised sample size of 40 participants in total across two research sites in the United Kingdom – Sheffield Teaching Hospitals National Health Service (NHS) Foundation Trust and University Hospitals of Leicester NHS Trust. The study recruited its first participant on 27^th^ March 2026. Recruitment is ongoing with a planned recruitment period of 12 months, estimated to complete enrolment by 9th February 2027. Participants will be recruited from specialist PDN clinics, and their aligned primary care and podiatry services. Recruitment will be supported through digital promotion, including social media, and via local patient groups and charities. All study investigations and interventions will take place in clinical research facilities at the two sites. Data collection is expected to complete by September 2027 with results expected to be shared by February 2028.

### Standard of care in both arms

HCL insulin therapy will be administered alongside standard care without requiring participants to stop their usual neuropathic pain medications. During the trial, minor adjustments to pain medication may occur at the clinician’s discretion to maintain safety and comfort, though further dose escalation is unlikely. All changes will be recorded and included in the analysis. Continuation of standard care is ethically and pragmatically justified, reflecting real-world practice, and facilitating recruitment as withholding standard neuropathic pain treatment would be unacceptable.

### Interventions

#### Control intervention period: CGM only (Dexcom G7).

In the control arm, participants will continue to use the insulin regimen on which they enter the study. As per NICE Quality Standard QS208, CGM is standard of care for pwT1D in the United Kingdom [21]. All participants will be provided with a Dexcom G7 continuous glucose monitoring (CGM) system. Standardisation of the CGM used enables comparison of glycaemic metrics between participants.

The Dexcom G7 is a CE-marked device that is worn on the back of the upper arm, requiring replacement every 10 days with a 30-minute warm up period [22]. Participants will download the Dexcom G7 app onto their smartphones to enable cloud-based data sharing. Participants will receive formal instruction as per the device manual on how to use the Dexcom G7. During control arm visits, a diabetes clinician will review participants, analyse their glucose data, and provide advice on optimising insulin treatment and glycaemic management. Regular pain medication will be continued throughout the study with changes to neuropathic pain medication avoided where possible. All medication changes will be documented.

#### HCL intervention period: HCL + CGM (t:slim X2 + Dexcom G7).

At the start of the HCL intervention period, participants will attend an in-person training visit for instruction of use of the Control-IQ and a Dexcom G7 CGM, based on manufacturer guidelines. The t:slim X2 is a tethered insulin pump licensed in the UK for use with quick acting insulins (Humalog, NovoRapid, Lyumjev, Admelog, and Trurapi) [23]. Control-IQ is an advanced HCL algorithm that uses predictive technology to automatically modulate insulin delivery. It reduces or suspends basal insulin to mitigate impending hypoglycaemia and increases basal insulin delivery and administers automated correction boluses to address impending hyperglycaemia. The system also includes temporary modes for sleep and exercise that adjust glycaemic targets, and allows for the creation of multiple insulin profiles to accommodate different situations, such as illness. Comprehensive education will be provided on sick-day management, including protocols for preventing diabetic ketoacidosis and instructions to switch to back-up insulin pens in the event of pump malfunction or infusion set failure.

The 12-week data collection period will commence after the training visit. All devices will be connected to the Tandem t:slim X2 mobile app to facilitate continuous cloud-based data upload. To ensure a safe transition to the new technology, a dedicated safety visit (Visit 3A) will be arranged shortly after the first scheduled infusion set change. At subsequent clinical visits, a diabetes clinician will review pump and CGM data, and provide advice to optimise pump settings. Concordance with hybrid closed loop treatment will be reviewed. The key therapeutic focus will be on improving glycaemic variability. To mitigate the risk of treatment-induced neuropathy of diabetes (TIND), a gradual reduction in average glucose levels will be implemented for participants with higher baseline HbA1c (>11.0%), by use of the pump in open loop mode for up to two weeks. A dedicated support number will be available for participants in all arms and sequences of the study. If ongoing treatment with HCL was deemed to be deleterious by the principal investigators, for example, due to worsening neuropathic pain, recurrent diabetic ketoacidosis, or any unforeseen recurrent or persistent issue, the trial participant would be returned to standard care.

#### Outcomes.

The primary outcome is the within-participant change in 7-day average 24-hour pain on an 11-point NRS from baseline to the end of each 12-week treatment period (sample pain diary in S2 File). A statistically significant reduction in the pain score will be considered to indicate superiority of the HCL treatment compared to standard care. This endpoint and measurement tool are considered the gold standard for clinical trials in PDN, aligning with the core domains of the IMMPACT consensus recommendations and NICE guidelines [18,24–26]. A 3-month endpoint is consistent with prior PDN studies, previously demonstrated to be a sufficient timeframe for clinical changes to be observed [27,28].

#### Secondary outcomes.

While the primary outcome addresses the core IMMPACT domain of pain intensity, secondary outcomes are included to capture the other recommended domains of physical and emotional functioning, as well as patient global impression of change (Table 2).

**Table 2:** Pre-specified secondary outcomes.

Pain-related outcomes
Proportion reaching 30% (moderate) and 50% (substantial) reduction in pain NRS
Change in area under the curve (AUC) for all daily NRS scores (12-week intervention period)
Changes in validated pain questionnaires: • Brief Pain Inventory (Modified Short Form) – BPI (MSF) • Neuropathic Pain Symptom Inventory (NPSI)
**Physical and emotional functioning**
Changes in validated questionnaires: • EQ-5D-5L • 36-Item Short Form Health Survey (SF-36) • Beck Depression Inventory (BDI) • Type 1 diabetes-specific distress (T1-DDS)
**Global impression of change**
Patient- and Clinician-rated Global Impression of Change (PGIC and CGIC)
**Glycaemic outcomes**
Key glycaemic metrics derived from CGM data, including time below range (TBR) <3.0 mmol/L, TBR 3–3.9 mmol/L, time in range (3.9–10 mmol/L), time in tight range (3.9–7.8 mmol/L), time above range >10–13.9 mmol/L, time above range >13.9 mmol/L, coefficient of variation (CV), standard deviation (SD), and Glucose Management Indicator (GMI)
Haemoglobin A1c (HbA1c)
**Safety outcomes**
The frequency of adverse and serious adverse events

NRS – Numerical Rating Scale. Time below range (TBR).

#### Exploratory endpoints.

Neurophysiological assessments will be performed to further understand the underlying mechanisms of painful diabetic neuropathy and the impact of glycaemic variability on these parameters. Sleep parameters and physical activity will be explored as an objective proxy of pain evolution and management. Validated hypoglycaemia questionnaires (HypoA-Q and Hypoglycaemia Confidence Scale (HCS)) will be completed at baseline and at the end of each treatment period to explore the relationship between hypoglycaemia and PDN.

#### Participant timeline and data collection.

A summary of key activities for study participants at each visit is summarised in Table 1. As per this schedule, some visits can be conducted remotely. Prior to participation, prospective participants will be provided with the participant information sheet and given the opportunity to ask questions (S3 File). Written informed consent will be obtained by the PI or delegate (S4 File). Re-consent will be obtained for any protocol amendments. 70% completion rate of pain and sleep diaries, and CGM data from the 4-week screening period will be required to be eligible for randomisation.

A delay of three months from end of the four-week baseline data collection to start of the HCL twelve-week intervention period is permitted to allow scheduling of group starts. Three visits are scheduled during each 12-week intervention period to facilitate titration of treatment. Participants will continue to complete daily pain and sleep diaries until the end of the study. At the conclusion of each treatment arm, participants will attend a final visit for comprehensive data collection, including validated questionnaires (as listed in the secondary outcomes), a blood test for haemoglobin A1c, and neurophysiological assessments.

Changes in level of participation within the trial are categorised in the following ways – no trial intervention, no trial related follow-up, and no further data collection – depending on the participant’s wishes. Where the participant consents, the participant will continue to attend the original schedule of assessments. Where they consent to data collection but do not wish to attend trial visits, where possible, data will be collected at standard clinic visits.

#### Randomisation.

Participants will be randomised at the level of the individual in a 1:1 ratio to either Treatment Sequence A (HCL + CGM followed by CGM only) or Treatment Sequence B (CGM only followed by HCL + CGM) using a secure online randomisation programme. The randomisation will use the Pocock-Simon minimisation algorithm to ensure balance in the treatment allocation taking into account centre, age (18–39, 40–59, 60+) and gender at birth [29].

#### Blinding (masking).

Given the nature of the HCL intervention and the use of a visible device, it is not possible to blind participants to their assigned treatment arm. To mitigate potential bias from research staff, pain surveys are completed solely by the participants, with other secondary outcome assessments such as neurophysiological assessments and haemoglobin A1c yielding objective numerical data, which are not subject to assessor bias.

#### Distress protocol.

Given that some secondary outcome questionnaires assess sensitive topics such as depression and quality of life, there is a potential for participants to experience psychological distress. To mitigate this risk, a formal distress protocol is in place (S5 File). This includes providing immediate support from the trial clinician and a pre-established pathway for onward referral to a specialist mental health team if necessary.

#### Neurophysiological tests.

Four objective point-of-care neurophysiological assessments will be completed at baseline and at the end of each treatment period. The CE- and FDA-approved Sudoscan device (Impeto Medical ltd, Issy-les-Moulineaux, France) will be used to assess small-fibre neuropathy, based on sudomotor function. The test is a rapid and reproducible non-invasive assessment [30]. Participants place their hands and bare feet on electrode plates for 3 minutes. A low-voltage current (<4V) is applied to stimulate small fibres innervating sweat glands, and based on the electrochemical reaction between sweat chloride and stainless-steel electrodes, the resultant electrochemical skin conductance (ESC) is measured [31]. As per previous data, an ESC of <70 µS for Caucasian individuals and <60µS for Asian and African American individuals is considered diagnostic of DPN, whereas an ESC above these thresholds is considered normal [32].

The CE- and FDA-approved DPN-check (NeuroMetrix Inc, MA, USA), a handheld device with stimulating probes at one end and a biosensor at the other will be used to assess for large-fibre neuropathy [30,31]. The sural sensory nerve conduction velocity (SNCV;/m/s) and amplitude (sural nerve action potential (SNAP)) will be measured in both ankles, taking approximately 2 minutes. The device has an in-built infrared thermometer to measure skin temperature near the ankle, with subsequent SNCV readings normalised for skin temperature. DPN is associated with low SNAP and SNCV. The proprietary thresholds cut-offs for diagnosis of DPN using the DPN-Check are a SNAP ≤ 4 µV and/or SNCV ≤ 40 m/s in at least one leg. The DPN-Check’s software also provides an objective classification of neuropathy severity based on the measured SNCV and SNAP values, categorising the findings into four stages: 0 (Normal), 1 (Mild DPN), 2 (Moderate DPN), and 3 (Severe DPN).

Vibration perception thresholds will be examined using multifrequency vibrometry via the VibroSense Meter II device (VibroSense Dynamics, Malmo, Sweden). The VibroSense Meter II tests vibration sensation at 4, 8, 16, 32, 64, 125 and 250 Hz at the right metatarsal head [33]. The examination is fully automated, with the vibration applied via a probe and the participant pressing down on a response button if they perceive vibration. Prior studies have demonstrated associations between deficits in vibration sensation and a higher risk of developing diabetic foot ulcers, gait or balance problems or weakness of the feet [34].

Autonomic function tests will be conducted with the Vagus Device (Medicus Engineering, Aarhus, Denmark), a handheld device, validated to diagnose autonomic neuropathy via two-electrode ECG recordings [35]. Assessment will comprise of two main components: time-domain and frequency-domain parameters from 5-minute resting heart rate variability; and four cardiac autonomic reflex tests (CARTs). The four standardised CARTs performed will be the expiration: inspiration (E:I) heart rate ratio during deep breathing, the heart rate response to a valsalva manoeuvre at 40 mmHg via a disposable mouthpiece, and both the heart rate and blood pressure response to standing. Results will be compared to published age- and sex-matched normative thresholds, with results below the 5^th^ percentile deemed abnormal [35].

#### Activity and sleep tracking.

Participants will be asked to use a Withings’ sleep tracker mat (Issy-les-Moulineaux, France) and an activity monitor watch during the study period. Sleep parameters collected will include total sleep time, sleep interruption, the apnoea-hypopnea index (AHI – a measure of sleep apnoea), nighttime heart rate and heart rate variability. Physical activity measures include active minutes, steps and skin temperature. These parameters will be used as exploratory objective proxy measures of pain evolution and management.

#### Sample size justification.

To detect a minimally important difference of 0.75 NRS points (between-patient SD of 2, within-participant SD = 1.44) with 90% power and two-sided α = 0.05, a crossover/pairwise comparison requires 39 evaluable participants. Allowing for 20% dropout during the eligibility screening period, we will recruit up to 49 participants. The 0.75-point NRS difference reflects a 15% reduction from a conservative baseline NRS of 5, consistent with IMMPACT guidelines which define a 15% between-group difference as clinically meaningful [18]. Calculations use the within-individual SD appropriate for crossover designs and therefore are conservative for detecting a true within-participant treatment effect. Using a lower baseline than typically seen in NeuP trials ensures the study is robustly powered, even in patients with milder pain (effect size 0.52). Our estimated dropout rates are conservative and allow for an increased dropout rate compared to previous HCL trials [14,16].

#### Data management.

Sheffield Teaching Hospitals NHS Foundation Trust has policies in place which are designed to protect the security, accuracy, integrity and confidentiality of Personal Data. The trial will be registered with the Data Protection Officer and will hold data in accordance with the Data Protection Act (2018 and subsequent amendments). Personal details for each participant taking part in the study will be linked to a unique identification number held locally on a study screening log in the investigator site file at each site. All participant-related reports and communication transferred to the sponsor will be identified by this identification number only. User accounts for Dexcom clarity, Glooko and Tandem Connect will be pseudonymised, using the study identification number and a fake date of birth. Database lock will take place once all data entries have been checked and sufficient verification processes are completed.

#### Reducing bias and ensuring objective assessment.

Although participants and clinicians cannot be blinded to HCL use, we will minimise bias through several measures. Pain and sleep diaries collected from intervention visits will be analysed by an assessor blinded to treatment allocation. Analyses will be conducted by a statistician blinded to treatment sequence (the treatment sequence will be coded). Outcomes include objective metrics (CGM, activity logs) alongside standardised daily pain and sleep diaries. A pre-specified analysis plan and crossover design further reduce bias, with each participant serving as their own control. Independent oversight will monitor data quality and interpretation.

#### Statistical analysis.

The primary analysis will be conducted using an intention-to-treat (ITT) approach, including all participants who were randomized. As a preliminary step prior to the primary analysis, the change in the coefficient of variation (CV) between the control intervention period and HCL intervention period will be evaluated to assess intervention effect on glycaemic variability. The primary analysis will proceed regardless of whether a statistically significant difference in glucose CV is observed. A linear mixed-effects model will be used to assess the primary outcome, with all analysis performed by a statistician blinded to the allocation of treatment sequences. The model will include treatment arm, period, sequence, gender at birth, centre and age group as fixed effects and participant as a random effect to account for the repeated measures design. Model assumptions will be assessed using residual versus fitted plots and Q-Q plots of residuals. The NRS pain score (0–10) will be analysed as a continuous outcome, consistent with established practice in chronic pain trials. If model assumptions are violated, sensitivity analyses using alternative modelling approaches will be considered. The primary hypothesis will be tested at a two-sided significance level of α = 0.05. To minimise the risk of bias associated with missing data, a multiple imputation approach will be used as the primary method for handling missing NRS scores. This analysis will include all randomised participants. A participant’s NRS score will be considered missing if fewer than five daily pain scores are recorded in the final seven days of the treatment period. Sensitivity analyses will be conducted, including a per-protocol analysis, to evaluate the robustness of the primary findings. Secondary outcomes will be analysed, comparing changes from baseline to the end of the treatment period, between the two treatment arms. Exploratory analyses will examine the impact of the HCL system on objective neurophysiological assessments (Sudoscan, DPN-Check, Vibrosense and Vagus) by correlating changes in glycaemic metrics with changes in neurophysiological data; and examine how change in a participant’s NRS score correlate with physical activity and sleep parameters.

#### Trial monitoring and management.

Study monitoring will be conducted by the trial sponsor, Sheffield Teaching Hospitals NHS Foundation Trust, as per standard operating procedures. Authorised representatives of the sponsor or an ethics committee may perform audits or inspections at the recruiting centres, including source data verification. The trial will be overseen by an independent Data Monitoring Committee (DMC), which will be responsible for safeguarding the interests of trial participants, assessing the safety and efficacy data, and monitoring the overall conduct of the study.

The reporting period for adverse events will be from the start of treatment until the end of trial follow-up. The recording and reporting of Adverse Events (AEs) will be in accordance with the UK Policy Framework for Health and Social Care Research, the Principles of Good Clinical Practice as set out in the UK Statutory Instrument (2004/1031; and subsequent amendments) and the requirements of the Health Research Authority (HRA).

#### Post-trial care.

At the last treatment visit, participants will be given advice on how to return to usual care. As per NICE TA943 [36], the majority of participants will be eligible for HCL continuation on the NHS. At present, NHS England plans to implement a roll-out of HCL to pwT1D meeting the eligibility criteria over 5 years [37]. Depending on the current guidance at the end of the study, HCL continuation will be facilitated wherever possible in collaboration with NHS services if the participant is eligible and has gained benefit from the treatment.

#### Patient and public involvement statement.

We thank the Lay Advice on Diabetes and Endocrine Research (LADDER) Panel in Sheffield, United Kingdom, for their invaluable input. The panel, which consists of patients, carers, and individuals with an interest in diabetes and endocrine conditions, have so far provided guidance on the study design, burden of interventions, and recruitment materials, with ongoing input planned for the future.

### Ethics and dissemination

The study will be conducted in accordance with the Declaration of Helsinki Ethical Principles for Medical Research involving Human Subjects and in accordance with the UK Policy Framework for Health and Social Care Research and applicable UK Acts of Parliament and Statutory Instruments (and relevant subsequent amendments), which include Data Protection Act 2018; and the Principles of Good Clinical Practice as set out in the UK Statutory Instrument (2004/1031; and subsequent amendments). Ethical approval was obtained from Health Research Authority, Health and Care Research Wales and the West Midlands – Edgbaston Research Ethics Committee on 17^th^ June 2025 (25/WM/0068). All participants will be provided with written information about the trial, including the procedures involved in the study before obtaining written informed consent. Any future non-substantial amendments will be managed by the sponsor and substantial amendments will be submitted to the Ethics Committee, with updates also made on the trial registry.

Study results will be communicated directly to trial participants and disseminated through conference presentations and peer-review publications. Authorship eligibility will be assessed strictly according to ICMJE guidelines.

## Discussion

The crossover design of this study allows for within-participant comparisons, reducing confounding and maximising data collection. By allowing each participant to serve as their own control, the study increases statistical power while ensuring ethical access to the HCL treatment for all study participants. Furthermore, the planned withdrawal of HCL in half of participants provides an opportunity to evaluate whether neuropathic pain returns to baseline levels following the intervention period.

Despite these strengths, certain limitations are inherent to the study design. Due to the interactive nature of HCL, blinding participants to their treatment sequence is not feasible. There is also a risk of attrition bias, as the order of treatment may influence retention. For example, participants randomised to the intervention first might be more prone to dropping out before the crossover phase.

TIND is a phenomenon in which rapid improvements in glycaemia can cause or accentuate painful diabetic neuropathy [38]. HCL has been demonstrated to achieve improvement in glycaemia [15], and a potential pitfall of the design is that a rapid improvement in glycaemia following HCL start might transiently worsen pain outcomes in some participants. To mitigate this risk, the protocol incorporates a two-week open loop period for participants with a high baseline HbA1c to ensure a more gradual transition. However, TIND could potentially act as a confounder or diluter of the primary outcome results.

## Conclusion

The PAINLESS study is the first to evaluate the impact of HCL initiation on pain intensity in pwT1D and PDN. Through a robust randomised, crossover design this study will also capture comprehensive patient-centred outcomes including physical function, emotional well-being, quality of life and activity. If successful, this intervention offers a transformative prospect for patients, alleviating debilitating pain by optimising the management of the diabetes itself.

## Supporting information

S1 File SPIRIT Checklist. The completed SPIRIT Checklist for the PAINLESS protocol.
(DOCX)

S2 File Pain and Sleep Diaries. The Pain and Sleep Diaries used in the PAINLESS study.
(DOC)

S3 File Participant Information Sheet (PIS). The Participant Information Sheet for the PAINLESS study.
(DOCX)

S4 File Consent form. The consent form used in the PAINLESS study.
(DOCX)

S5 File Distress Protocol – The protocol detailing the arrangements for managing potential distress that may be experienced by participants while completing questionnaires during research visits. (DOCX)

S6 File Ethics board approved Protocol. (DOCX)
