# Supplementary material for: Pain alleviation in diabetic neuropathy using hybrid closed loop insulin pumps (PAINLESS) – A randomised crossover trial protocol
Source: PLoS One. 2026 Sep 8;21(9):e0357573. doi: 10.1371/journal.pone.0357573 (PMC13552766; doi:10.1371/journal.pone.0357573)
Supplement: Supplementary file 2 [file pone.0357573.s003.docx]

**Participant Information Sheet**

**PAINLESS STUDY**

Pain Alleviation In diabetic Neuropathy using hybrid cLosEd Loop inSulin pumpS: PAINLESS study

1. Invitation and brief summary

# We would like to invite you to take part in a clinical trial (research study). We are looking at whether reducing blood glucose variability can reduce pain from the nerve damage caused by diabetes.

# Joining this study is completely voluntary and your decision will not affect your standard of care in any way. Before you decide we would like you to understand why we are doing this research and what it will involve. Please take your time to read through this information carefully. Discuss it with your friends and family if you wish.

# This information sheet tells you why we are doing the study, what will happen to you if you take part and detailed information about how it will be run. Please do get in touch with us if you have any questions or any of the information is unclear.

2. Why are we doing this research

# Peripheral neuropathy is a common complication of diabetes and happens because of nerve damage in the feet and legs. Patients with neuropathy may have pain, discomfort or loss of feeling in the areas affected. The pain caused by peripheral neuropathy can badly affect sleep and quality of life.

# The standard treatment for nerve pain is medication but this doesn’t always work in some patients and there can also be side effects and problems tolerating the drugs.

# In this trial we are going to test hybrid closed loop (HCL) insulin pump treatment in adults with Painful Diabetes-Related Peripheral Neuropathy (PDPN). We hope that reducing blood glucose variability using HCL treatment will reduce pain and improve sleep quality and overall quality of life.

# In this cross-over trial participants will be randomly allocated to one of two treatment sequences:

- Treatment sequence A: HCL and DEXCOM G7 continuous glucose monitoring followed by DEXCOM G7 continuous glucose monitoring only
- Treatment sequence B: DEXCOM G7 continuous glucose monitoring only followed by HCL and DEXCOM G7 continuous glucose monitoring.

# The rest of your treatment will continue as normal.

**Figure 1: Hybrid closed loops insulin treatment used in PAINLESS STUDY**

3. What are the treatments?

What is HCL insulin pump?

Hybrid closed-loop insulin treatment is a system that helps people with diabetes manage their blood sugar levels more easily. It combines an insulin pump and a continuous glucose monitor (CGM) to automatically adjust the amount of insulin delivered to the body.

Here’s how it works:

- Insulin pump: This device provides insulin to the body throughout the day.
- Continuous glucose monitor (CGM): This sensor measures blood sugar levels continuously, sending the data to the insulin pump.

The system uses these glucose readings to adjust insulin delivery automatically, keeping blood sugar in a healthy range most of the time. It's called "hybrid" because, while it automates a lot, the user still needs to enter information at certain times, like meal details, to fine-tune insulin doses.

In short, the hybrid closed-loop system makes life with diabetes easier by automating some of the insulin dosing, but the user still has to be involved in managing their treatment, especially around meals.

You can continue all usual activities while using hybrid closed-loop systems and CGM including exercise, swimming, bathing and sports. We will give you instructions on how to manage the technology during the study.

4. Why have I been invited?

# We are inviting people who have had type 1 diabetes for more than 5 years, and who have had daily neuropathic pain for at least 3 months despite taking medication for it. As described in section 2, we hope that the treatment we are studying will reduce the pain that people with painful neuropathy experience.

5. What would taking part involve?

SCREENING VISIT

# At this face-to-face visit you have the opportunity to ask any questions you might have. You will then be given some time to think about what you would like to do. If you decide you would like to participate you will be asked to sign and date a consent form. The doctor will also sign this and give you a copy.

# During this first visit you will be asked some questions about your medical history and a clinical exam including simple nerve tests that will be done by your doctor. This is to help the research team check you are eligible to take part. You will be asked to complete a set of questionnaires about your pain, sleep, mood and general health and wellbeing. The nature of some of the questions asked may be sensitive and include questions about depression and thoughts of suicide. You can take a break at any time, and the interview can be stopped if you do not want to continue. Should your answers reflect suicidal tendencies the research team has a duty of care to make your clinical care team aware.

# Your blood pressure, height and weight will be checked. You may also be asked to give a blood sample depending on when the last one for your diabetes was taken. At the end of the visit, if after answering the questions, the research team find that you are eligible to take part, you will be fitted with a DEXCOM G7 continuous glucose sensor, given sleep and activity trackers and will be asked to complete a 7-day pain diary for 4 weeks. If you are not eligible to take part, we will explain the reasons and you will not continue in the study.

RANDOMISATION TO A TREATMENT

# At the next visit, the research team will check the glucose data and the pain diaries to confirm your eligibility to continue. Once all screening and baseline procedures have been done and eligibility has been confirmed by your doctor you will be randomised using an online system. Neither you, your clinical care team nor the research team will have any control over who receives which treatment. If you are randomised to Treatment Sequence A (i.e. to receive HCL insulin pump treatment during the first period), we will arrange a visit to commence HCL insulin pump treatment with a study nurse. These sessions will be performed in groups of maximum four patients. Three days after starting HCL insulin pump treatment, our research team will contact you over the phone to ensure the HCL pump is working okay and troubleshoot any issues. The next visit will be 2 weeks later. During this face-to-face visit we will optimise the pump settings and provide you with new pain diaries. The subsequent 2 study visits will be conducted either virtually or face-to-face every 4 weeks to review glucose data and to optimise HCL pump settings. The final study visit (12 weeks after starting on the HCL pump) will be performed face-to-face, where the same procedures performed during the Screening Visit will be repeated. At the end of this visit the HCL insulin pump treatment will be discontinued and your usual insulin treatment we be restarted.

# If you are randomised to Treatment Sequence B, you will continue using the DEXCOM G7 continuous glucose monitor and study visits with the research team will be booked every 4 weeks to review your blood glucose data and optimise your insulin treatment. Depending on your preferences, these visits can be performed either virtually or face-to-face. 16 weeks after the initial Screening Visit, we will ask you to attend for a face-to-face visit, where the same procedures performed during the Screening Visit will be repeated. At the end of this study visit, you will be given an appointment to commence HCL insulin pump treatment and continue on HCL for 12 weeks as described above.

PAIN DIARY

We will ask you to keep a pain diary, every day for 6 months. You will do this in the same way as during the screening part of the trial using a paper diary.

| **Visit** |  | **What will happen at the visit** | **Time duration** |
| --- | --- | --- | --- |
| **1: Screening** | IP | Discuss the study, asked to sign consent  Medical history  Questionnaires  Tests of nerve function  Blood test for HbA1c (if not done in last 3 months)  Dexcom G7 CGM fitted and instructions given  Advised on pain and sleep diaries  Given sleep and activity tracker | 90-120 min |
| **2: Randomisation** | IP | Eligibility confirmed (dependent on CGM, pain and sleep diaries)  Randomised to treatment sequence | 30 min |
| ***Depending on your randomised sequence, you will start at HCL Pump Start or 3B. Following completion of visits 7A or 6B respectively, you will switch to the other set of visits*** | | | |
| **HCL Pump Start** | IP | Group session  Instructions on how to use the pump | 180-240 min |
| **3A** | T | Check-in to see if pump working, troubleshooting | 30 min |
| **4A** | IP | Review glucose data, optimise pump settings | 30 min |
| **5A** | T/IP | Review glucose data, optimise pump settings | 30 min |
| **6A** | T/IP | Review glucose data, optimise pump settings  Given pain diaries | 30 min |
| **7A** | IP | Pain diaries collected  Download glucose and pump data  Blood test for HbA1c  Questionnaires  Tests of nerve function  Instructed on how to switch back to usual treatment | 90 min |
| **3B** | T | Review glucose data, optimise insulin treatment | 30 min |
| **4B** | T/IP | Review glucose data, optimise insulin treatment | 30 min |
| **5B** | T/IP | Review glucose data, optimise insulin treatment  Given pain diaries | 30 min |
| **6B** | IP | Pain diaries collected  Download glucose and pump data  Blood test for HbA1c  Questionnaires  Tests of nerve function | 90 min |

***Table 1:*** Information on visits. IP – In-person; T – Telephone.

6. What are the possible benefits of taking part?

You will receive a thorough check of your condition and could try the new HCL insulin pump therapy before it is more widely available. While there may be no immediate benefits to you, the aim is to improve care for people with pain from their diabetes.

7. What are the possible disadvantages and risks of taking part?

# Study visits and completing the questionnaires can be time consuming but we have kept study visits to a minimal and most will be performed virtually. There are no major side effects expected from either treatment arms. Whilst changing from injections to a pump or back again, you may experience changes in glucose control. The research team will support you through this period and give you advice on how to change your doses. If there are any major concerns, you will be able to contact the research team.

8. Who is organising and funding the research?

# The study is sponsored by Sheffield Teaching Hospitals NHS Foundation Trust, which means it has certain legal and ethical responsibilities for the study. It is being funded by TANDEM Diabetes, the manufacturer of HCL insulin pumps that will be used in this study. The chief investigator for the trial is Dr Dinesh Selvarajah, based at the University of Sheffield.

9. Who has reviewed the study?

# All research which takes part in the NHS is looked at by an independent group of people who protect your safety, rights, wellbeing and dignity. This group is called a Research Ethics Committee. Before we asked any patients to join, the trial was reviewed and given a favourable opinion by the West Midlands – Edgbaston Research Ethics Committee.

10. Who is funding the research?

This study is funded by TANDEM Diabetes. The views expressed are those of the author(s) and not necessarily those of TANDEM Diabetes.

11. Involvement of your usual care givers

# We will tell your GP and your diabetes specialist that you are taking part in the study.

12. Will my taking part in this study be kept confidential?

# Yes. Your participation in the study and all information collected will be subject to the General Data Protection Regulation and the Data Protection Act (2018) and will be kept strictly confidential. Each participant will be given a unique study specific code number that will be used to identify them on study documentation/records, this is called pseudonymisation.

13. How will we use information about you?

# We will need to use information from you and your medical records for this trial. This information will include:

| **When** | **What data will be collected** |
| --- | --- |
| Screening visit | - Full name (first, middle & surname) - Email address - Mobile phone number (for virtual study visits) - Full date of birth, gender, NHS number - Contact details: email address and/or mobile phone number - Relevant medical history - Current medications you are taking - Physical exam and questionnaires completed during this visit |
| Follow-up visits | - Blood glucose readings to optimise insulin treatment |
| End of treatment visits | - Questionnaires completed during this visit - Current medication and medical history - Physical exam |

# People will use this information to do the research or to check your records to make sure that the research is being done properly. People who do not need to know who you are will not be able to see your name or contact details. Your data will have a code number instead i.e. a pseudonymised participant ID number. This pseudonymised data may be shared with the University of Sheffield for purposes of data analysis.

# Sheffield Teaching Hospitals NHS Foundation Trust is the sponsor of this research and is responsible for looking after your information. We will keep all information about you safe and secure by:

# Storing any physical paperwork containing identifiable data in an access-controlled and secured rooms.

# Storing electronic data on secure, encrypted IT servers.

# Once we have finished the trial, we will keep some of the data so we can check the results. We will write our reports in a way that no-one can work out that you took part in the study.

# We will keep your study data for 15 years after the end of the study. The study data will then be fully anonymised and securely archived or destroyed.

# You can stop being part of the study at any time, without giving a reason, but we will keep information about you that we already have.

# If you choose to stop taking part in the study, we would like to continue collecting information about your health from central NHS records, your hospital or your GP. This is optional. We will ask you for your consent to opt-in to ongoing collection of information for the duration of the study.

# You have the right to ask us to remove, change or delete data we hold about you for the purposes of the study. We might not always be able to do this if it means we cannot use your data to do the research. If so, we will tell you why we cannot do this.

**Where can I find out more about how my information is used?**

# You can find out more about how we use your information, including the specific mechanism used by us when transferring your data.

- our leaflet: https://www.sheffieldclinicalresearch.org/for-patients-public/how-is-your-information-handled-in-research/
- by asking one of the research team
- by sending an email to the Sponsor’s Data Protection Officer at [sth.InfoGov@nhs.net](mailto:sth.InfoGov@nhs.net), or
- by ringing the Sponsor’s Data Protection Officer on 0114 2265151
- in the leaflet available from [www.hra.nhs.uk/patientdataandresearch](http://www.hra.nhs.uk/patientdataandresearch)

14. What if something goes wrong?

# We do not expect any problems as a result of your participation in the trial. However, all patients are covered for negligent harm according to NHS indemnity guidelines. If you have a concern about any part of this trial, you should ask to speak to a member of the research team who will do their best to answer your questions. If you are harmed by your participation in this study and this is due to someone’s negligence then you may have grounds for a legal action for compensation against <insert Trust name>, but you may have to pay your legal costs.

# If you wish to complain about any aspect of the way you have been approached or treated during this trial, the normal NHS complaints mechanisms will be available to you, and you are not compromised in any way because you have taken part in a research study. Should you have any cause for concern about the running of the study please contact the Chief Investigator Dr Dinesh Selvarajah by email: [d.selvarajah@sheffield.ac.uk](mailto:d.selvarajah@sheffield.ac.uk). If you remain unhappy and wish to formally complain, please contact the Patient Advice and Liaison Service (PALS) or the Complaints Team at your local hospital.

*<NHS Patient Advisory and Liaison Service (PALS); or local equivalent>*

Tel: *<insert local PALS contact number(s)>* Email: *<insert local PALS email address>*

15. What if I do not want to take part?

# Participation in the study is entirely voluntary and if you decide not to take part you do not have to give a reason for this. If you do choose to enter the study, you are free to change your mind and withdraw at any point.

# The standard of care provided to you by your doctor will not be affected in anyway should you decide not to participate.

16. Will my travel expenses be reimbursed?

# Yes, we can help towards your travel expenses for trial visits to the hospital. Travel costs of up to £25 per visit can be reimbursed. Please speak to your research team about this who will be happy to arrange for you. In this study, there is no provision for payment for your time spent in the study.

17. What happens if new information becomes available?

# Sometimes we get new information about the treatment being studied. If this happens, a member of the research team will tell you and discuss whether you should continue in the study. If your research doctor is happy for you to continue in the study, you will have the option to decide whether to continue. A member of the research team may ask you to re-sign a consent form if you do.

# If you decide not to carry on, a member of the research team will make arrangements for your standard clinical care to continue.

# Sometimes your study doctor might consider it to be in your best interest to withdraw you from the trial. If this is the case, they will explain their reasons for this and arrange your continuing care.

# If for any reason the study is stopped, you will be informed and your continuing care arranged.

18. What happens when the research study stops?

# At the end of the study, your usual doctor will continue to look after you and your treatment.

19. Can I keep my study devices after the study has finished?

At the end of the study, devices will need to be returned. However, if you have gained benefit from the devices and you are eligible on NHS, we will liaise with your local team to facilitate continuation on equivalent technology where possible and ask them to take over ordering of supplies.

20. What will happen to the results of the research study?

# Once the study is completed and the data is collected and analysed, we will publish our results in medical journals and present them at scientific meetings, to help other doctors to learn, and patients to benefit. The study findings will also be shared with you as a participant and made available to patients and the public through patient organisations, health information websites that are open to the public and the media. It will not be possible to identify you in any of these reports, the data will be presented in an anonymous manner.

21. Do you have any further questions?

# You will have some time to think about the study and make your decision. You may wish to discuss it with your family or friends. If you take part, you will receive a copy of this information sheet and a copy of the signed Consent Form to take home. If, at any time, you have any questions about the study you should contact your study doctor or research nurse using the details below.

# Thank you for taking the time to read this information sheet.

**Contact Information**

If you would like to speak to the PAINLESS Study team about the study, please contact:

*< Contact Name > <Job Title> <Telephone and/or E-mail>*
