# Supplementary material for: Pain alleviation in diabetic neuropathy using hybrid closed loop insulin pumps (PAINLESS) – A randomised crossover trial protocol
Source: PLoS One. 2026 Sep 8;21(9):e0357573. doi: 10.1371/journal.pone.0357573 (PMC13552766; doi:10.1371/journal.pone.0357573)
Supplement: Supplementary file 3 [file pone.0357573.s004.docx]

**PAINLESS Study**

Pain Alleviation In diabetic Neuropathy using hybrid cLosEd Loop inSulin pumpS: PAINLESS study

**Informed Consent Form**

**Version 1.1 08-05-2025**

**Principal Investigator:** ______________________________

**PAINLESS Study Number**

| 1 | I confirm that I have read and understood the **PAINLESS STUDY** Participation Information Sheet version number __. __ dated _ _ /_ _ _ / _ _ _ _. I have also had enough opportunity to think about the information provided in information sheet and ask questions and have had these answered satisfactorily. |  |
| --- | --- | --- |
| 2 | I understand that my participation in this trial is entirely voluntary and I am free to withdraw from screening at any time without giving any reason and without affecting my legal rights or the quality of my medical care. |  |
| 3 | I understand that for the purpose of the trial relevant sections of my medical notes might be looked at by responsible individuals from the **PAINLESS STUDY** research team, the sponsor (Sheffield Teaching Hospitals NHS Trust), or regulatory authorities to check if I meet the criteria to participate in the main **PAINLESS STUDY** and to check that the screening process is carried out correctly. |  |
| 4 | I agree to complete the pain diary as described in the Participant Information Sheet to participate in the **PAINLESS STUDY**. |  |
| 5 | Data collected that identifies me by name, will be transferred to the central organisers Sheffield Teaching Hospitals NHS Trust where it will be held securely and confidentially. I give permission for the transfer and storage of this data. |  |
| 6 | I agree to my GP and Hospital Diabetes Consultant being informed of my participation in the study and for the details of the information I provide to be checked with my GPs and/or hospital records. |  |
| 7 | I understand that all data collected will be used for medical research purposes only and that I will not be identified in the analysis or reporting of any results. |  |
| 8 | I understand that if I do not complete the study, this consent form and my data collected during the screening process will not be deleted but will not be shared or used for the trial in any way. |  |
| 9 | **OPTIONAL**: If I stop taking part in the study, I consent to the research team continuing to collect information from my NHS record, hospital diabetes team and GP for the duration of the study. I understand that I can withdraw my consent for this at any point. | **Yes**  **No** |
| 10 | **OPTIONAL:** I wish to receive a copy of this consent form via email.  If you select ‘Yes`, please provide the email address below that you wish the consent form to be sent to.  Email…………………………………………………………………………………… | **Yes**  **No** |
| 11 | **I voluntarily agree to take part in the PAINLESS STUDY.** |  |

| **Participant’s full name** | **Participant’s signature** | **Please confirm the date you, the participant signed this form:** |
| --- | --- | --- |
|  |  | D  D  M  M  Y  Y  Y  Y  M |

**Researcher taking consent**: I confirm that the nature of the PAINLESS STUDY has been explained to the above named patient.

| **Researcher’s full name** | **Researcher’s signature** | **Please confirm date researcher signed this form:** |
| --- | --- | --- |
|  |  | D  D  M  M  Y  Y  Y  Y  M |

**This consent form is now complete.**

This study is funded by TANDEM Diabetes Care (project reference xx). The views expressed are those of the authors and not necessarily those of TANDEM Diabetes Care.
