# Supplementary material for: Pain alleviation in diabetic neuropathy using hybrid closed loop insulin pumps (PAINLESS) – A randomised crossover trial protocol
Source: PLoS One. 2026 Sep 8;21(9):e0357573. doi: 10.1371/journal.pone.0357573 (PMC13552766; doi:10.1371/journal.pone.0357573)
Supplement: Supplementary file 4 [file pone.0357573.s005.docx]

**PAINLESS Study**

**Distress Protocol**

Arrangements for managing potential distress that may be experienced by participants while completing questionnaires during research visits.

**All questionnaires** will be screened **immediately** following completion to check their content for any relevant areas of clinical concern. This screening will be done by the research team member at the research visit.

Distress experienced by participant

Pause research visit and check-in with participant

Continue research visit

Stop research visit

Active suicidal ideation identified

Debrief and remind participant about support available

Same day referral to local emergency mental health team e.g. crisis team

Researcher informs Principal Investigator that distress was experienced

Routine follow-up call and additional support offered

If distress too high to be managed, referral to appropriate NHS services via GP and/or usual care team as appropriate

No additional support needed

**Arrangements for managing potential distress that may be experienced by the researcher:**

The research team will also have access to support structures in place within their organisation.

**Additional Information**

**If active suicidal ideation is uncovered**, a member of the research team will gain verbal consent from the participant to discuss these matters with the relevant member of their usual care givers, for example, their GP or hospital diabetes specialist. An assessment of the risk will be performed, and if appropriate, a same-day referral to the local emergency mental health team (e.g. crisis team) will be made. The matter should be discussed urgently with the principal investigator or nominated deputy.

**If other mental health concerns are uncovered, with no active suicidal ideation**, a member of the research team will gain verbal consent from the participant to discuss these matters with the relevant member of their usual care givers, for example, their GP or hospital diabetes specialist. An assessment of the risk will be performed. If no immediate risk is identified, their usual caregivers will be asked to follow-up the concern and refer along usual local pathways. Participants will be signposted to self-referral services such as talking therapies (Vita mind in Leicester, Sheffield talking therapy in Sheffield) where appropriate.

**Contact details for services:**

**Sheffield Health and Social Care Crisis Team:**

https://www.shsc.nhs.uk/services/urgent-and-crisis-service

0114 226 3636 or 0808 1968281 or via 111 and choosing the mental health option (24/7)

**Leicester crisis team**

<https://www.leicspart.nhs.uk/service/central-access-point/>

0300 300 1010 or via 111 and choosing the mental health option (24/7)

**Sheffield Talking Therapies**

<https://www.sheffieldtalkingtherapies.nhs.uk/>

[0114 226 4380](tel:01142264380) (available Monday to Friday 8.30am – 5pm)

**InVita Talking Therapies (Leicester)**

https://www.vitahealthgroup.co.uk/nhs-services/nhs-mental-health/leicester-leicestershire-rutland/

0330 094 5595 (Monday to Thursday 8am – 8pm Friday 8am- 5.00pm)
