# Supplementary material for: Pain alleviation in diabetic neuropathy using hybrid closed loop insulin pumps (PAINLESS) – A randomised crossover trial protocol
Source: PLoS One. 2026 Sep 8;21(9):e0357573. doi: 10.1371/journal.pone.0357573 (PMC13552766; doi:10.1371/journal.pone.0357573)
Supplement: Supplementary file 5 [file pone.0357573.s006.docx]

**TRIAL PROTOCOL**

**PAINLESS STUDY**

Pain Alleviation In diabetic Neuropathy using hybrid cLosEd Loop inSulin pumpS: PAINLESS study

This protocol has regard for the HRA guidance and is compliant with the SPIRIT guidelines (2013)

| **Version Number:** | **V1.1** |
| --- | --- |
| **Version Date:** | 04-Dec-2025 |

| Funding and support in kind |  |
| --- | --- |
|  | Financial and non-financial support given: |
| TANDEM Diabetes Care | US$111,361  44 Tandem T-slim pumps with Control IQ and consumables (124 infusion sets and reservoir/cartridges) for 6 months. |
| DEXCOM | Sensors for 40 patients for 6 months. A total of 900 sensors. |
| Withings | Sleep sensors for 40 patients. |
| VibroSense | VibroSense Meter II. One device per study site |
| The Funder of the trial has had no role in the trial design, data collection, data analysis or data interpretation. The views expressed are those of the authors and not necessarily those of TANDEM Diabetes or DEXCOM. | |

| Chief Investigator (CI) signature page | |
| --- | --- |
| As Chief Investigator, I confirm that I have read and agree with the following protocol, and that I will conduct the trial in compliance with the version of this protocol approved by the REC and any other responsible organisations.    I agree to ensure that the information contained in this document will not be used for any purpose other than the evaluation or conduct of the clinical investigation without the prior written consent of the Sponsor.  I also confirm that I will make the findings of the trial publicly available through publication or other dissemination tools without any unnecessary delay and that an honest accurate and transparent account of the study will be given; and that any discrepancies from the study as stated in this and any subsequent approved protocol will be explained. | |
| Trial name: | PAINLESS Study |
| Protocol version number: | Version: 1.1 |
| Protocol version date: | 04 Dec 2025 |
|  | |
| CI name: | Dr Dinesh Selvarajah |
| Signature and date: | ______________04/Dec/2025______ |
|  | |
| Sponsor statement  Where the Sheffield Teaching Hospitals NHS Trust takes on the sponsor role for protocol development oversight, the signing of the IRAS form by the sponsor will serve as confirmation of approval of this protocol. | |
| Compliance statement  This protocol describes the PAINLESS Study only. The protocol should not be used as a guide for the treatment of people not taking part in the PAINLESS Study.  The trial will be conducted in compliance with the approved protocol, the UK Policy Framework for Health and Social Care Research, Data Protection Act 2018 and the Principles of Good Clinical Practice (GCP) as set out in the UK Statutory Instrument (2004/1031) and subsequent amendments thereof.  Every care has been taken in the drafting of this protocol, but future amendments may be necessary, which will receive the required approvals prior to implementation. | |

| Principal Investigator (PI) signature page | |
| --- | --- |
| As Principal Investigator, I confirm that the following protocol has been agreed and accepted, and that I will conduct the trial in compliance with the approved protocol where this does not compromise participant safety.  I agree to ensure that the information contained in this document will not be used for any other purpose other than the evaluation or conduct of the clinical investigation without the prior written consent of the Sponsor. | |
| Trial name: | PAINLESS Study |
| Protocol version number: | Version: 1.1 |
| Protocol version date: | 04 Dec 2025 |
|  | |
| PI name: |  |
| Name of Site: |  |
| Signature and date: | _________________________ __ __ / __ __ / __ __ __ __ |

ADMINISTRATIVE INFORMATION

| Reference Numbers |  |
| --- | --- |
| Sponsor number | STH23249 |
| ISRCTN reference number |  |
| IRAS reference number | 352762 |

| Sponsor |  |
| --- | --- |
| Sheffield Teaching Hospitals NHS Trust | **Clinical Research & Innovation Office**,  Sheffield Teaching Hospital NHS Foundation Trust  D Floor, Royal Hallamshire Hospital  Glossop Road, Sheffield, S10 2JF |
| Contact details:  Dr Erica Wallis | [erica.wallis1@nhs.net](mailto:erica.wallis1@nhs.net) |

| Chief Investigator |  |
| --- | --- |
| Dr Dinesh Selvarajah | Senior Lecturer in Diabetes |
| Division of Clinical Medicine  Medical School  University of Sheffield  Beech Hill Road  S10 2RX | Mobile: 07747175373  [d.selvarajah@sheffield.ac.uk](mailto:d.selvarajah@sheffield.ac.uk) |

| Co-investigator Group |  |
| --- | --- |
| Dr Ahmed Iqbal | Senior Lecturer in Diabetes, University of Sheffield |
| Dr Simon Berry | Clinical Research Fellow, University of Sheffield |
| Dr Gordon Sloan | Clinical Lecturer, University of Sheffield |
| Prof Solomon Tesfaye | Consultant Diabetologist, Sheffield Teaching Hospitals NHS Trust |
| Dr Jackie Elliott | Consultant Diabetologist, Sheffield Teaching Hospitals NHS Trust |
| Prof Pratik Choudhary | Prof of Diabetes, University of Leicester |

ABBREVIATIONS

| Abbreviation | Term |
| --- | --- |
| AE | Adverse Event |
| AUC | Area Under the Curve |
| BDI | Beck’s Depression Inventory |
| BPI | Brief Pain Inventory |
| CGIC | Clinician Global Impression of Change 7 item Likert Scale |
| CGM | Continuous Glucose Monitoring |
| CI | Chief Investigator |
| CRF | Case Report Form |
| DCF | Data Clarification Form |
| DM | Diabetes Mellitus |
| DN4 | Douleur Neuropathique en 4 questions |
| EQ-5D-5L | EuroQol Five Dimensions Five Levels |
| GCP | Good Clinical Practice |
| GV | Glycaemic Variability |
| HbA1c | Haemoglobin A1c - Measurement of glucose in haemoglobin |
| HCL | Hybrid Closed Loop |
| HRA | Health Research Authority |
| HypoAQ | Hypoglycaemic Awareness Questionnaire |
| ICF | Informed Consent Form |
| ISF | Investigator Site File |
| NHS | National Health Service |
| NICE | National Institute for Health and Care Excellence |
| NPSI | Neuropathic Pain Symptom Inventory |
| NRS | Numerical Rating Scale |
| PGIC | Patient Global Impression of Change 7 item Likert Scale |
| PI | Principal Investigator |
| PIS | Participant Information Sheet |
| PPI | Patient and Public Involvement |
| RCT | Randomised Controlled Trial |
| REC | Research Ethics Committee |
| SAE | Serious Adverse Event |
| SF-36 | Short Form-36 |
| T1-DDS | Type 1 Diabetes Distress Score |
| TCNS | Toronto Clinical Neuropathy Score |
| TMF | Trial Master File |
| VAS | Visual Analogue Scale |

TRIAL SUMMARY

Title

Pain Alleviation In diabetic Neuropathy using hybrid cLosEd Loop inSulin pumpS: PAINLESS study

Aim

The aim of the trial is to evaluate the impact of reducing glycaemic variability using Hybrid Closed Loop (HCL) insulin pumps on pain intensity compared to control in painful diabetic neuropathy.

Primary Objective

To evaluate the impact of reducing glycaemic variability using HCL insulin pumps on pain intensity compared to control in painful diabetic neuropathy.

Trial design

Two-centre, randomised, cross-over, feasibility study.

Participant population and sample size

40 adults with painful diabetic neuropathy

Setting

Two teaching, NHS Hospital Trusts.

Eligibility criteria

Inclusion criteria:

- Clinical diagnosis of type 1 diabetes for at least 5 years
- Daily neuropathic pain for at least 3 months,
- 24-hr average pain score > 4 (BPI-MSF),
- Hba1c between 6.5 to 13%,
- No change to dose of neuropathic pain medication within the last month,

Exclusion criteria:

1. Non-diabetic neuropathies,
2. Other chronic pain disorders more severe than painful diabetic neuropathy,
3. History of major psychiatric disorders,
4. Pregnancy/breast feeding or planning pregnancy during the course of the study.

Interventions

HCL+CGM vs CGM only

Primary Outcome

Change in pain as measured by the 7-day average 24-hour pain on an 11-point NRS scale (0 = no pain and 10 = worst pain imaginable) during the final follow-up week (3 months post-randomisation).

CONSORT DIAGRAM

TRIAL FLOW DIAGRAM

CONTENTS

[1.	BACKGROUND AND RATIONALE	14](#_Toc185254331)

[1.1.	Background	14](#_Toc185254332)

[1.2.	Justification for design	14](#_Toc185254333)

[1.3.	Justification for participant population	15](#_Toc185254334)

[1.4.	Justification of choice of intervention	15](#_Toc185254335)

[1.4.1	HCL vs. regular insulin pumps vs MDI	16](#_Toc185254336)

[1.5.	Justification of choice of primary outcome	17](#_Toc185254337)

[2.	AIMS AND OBJECTIVES	17](#_Toc185254338)

[2.1.	Main trial objectives	17](#_Toc185254339)

[2.1.1	Clinical objectives	17](#_Toc185254340)

[2.1.2	Feasibility assesments	17](#_Toc185254341)

[2.1.3	Safety objectives	18](#_Toc185254342)

[2.1.4	Exploratory objectives	18](#_Toc185254343)

[3.	TRIAL DESIGN AND SETTING	18](#_Toc185254344)

[3.1.	Assessment of risk	18](#_Toc185254345)

[4.	ELIGIBILITY	19](#_Toc185254346)

[4.1.	Inclusion criteria	19](#_Toc185254347)

[4.2.	Exclusion criteria	19](#_Toc185254348)

[4.3.	Co-enrolment	19](#_Toc185254349)

[5.	CONSENT	19](#_Toc185254350)

[5.1.	Study consent	19](#_Toc185254351)

[5.2.	Randomisation	20](#_Toc185254352)

[6.	IDENTIFICATION, RANDOMISATION and BLINDING	20](#_Toc185254353)

[6.1.	Identification	20](#_Toc185254354)

[6.2.	Participant Identification Centres	21](#_Toc185254355)

[6.3.	Screening	21](#_Toc185254356)

[6.4.	Randomisation	21](#_Toc185254357)

[6.4.1	Randomisation method	21](#_Toc185254358)

[6.5.	Blinding	22](#_Toc185254359)

[6.6.	Informing the participant’s GP and other parties	22](#_Toc185254360)

[7.	TRIAL INTERVENTION	22](#_Toc185254361)

[7.1.	Trial intervention and dosing schedule	22](#_Toc185254362)

[7.2.	Interactions or contraindications	22](#_Toc185254363)

[7.2.1	Concomitant medication(s)/intervention(s)	22](#_Toc185254364)

[7.2.2	Prohibited medication(s)/intervention(s)	22](#_Toc185254365)

[7.3.	Post-trial care	22](#_Toc185254366)

[8.	OUTCOME MEASURES	23](#_Toc185254367)

[8.1.	Main trial outcomes	23](#_Toc185254368)

[8.2.	Primary outcome	23](#_Toc185254369)

[8.3.	Secondary Outcome	23](#_Toc185254370)

[8.3.1	Clinical	23](#_Toc185254371)

[8.3.2	Safety	23](#_Toc185254372)

[8.4.	Exploratory Endpoint	23](#_Toc185254373)

[9.	TRIAL PROCEDURES	23](#_Toc185254374)

[9.1.	Schedule of assessments	26](#_Toc185254375)

[9.2.	Withdrawal and changes in levels of participation	28](#_Toc185254376)

[10.	ADVERSE EVENTS REPORTING	28](#_Toc185254377)

[10.1.	Definitions	28](#_Toc185254378)

[10.2.	Adverse event recording – general	29](#_Toc185254379)

[10.3.	AE reporting in PAINLESS STUDY	29](#_Toc185254380)

[10.4.	SAE reporting in PAINLESS STUDY	30](#_Toc185254381)

[10.4.1	SAEs not requiring reporting to the Trial Office	30](#_Toc185254382)

[10.4.2	SAEs requiring non-expedited reporting to the Trial Office	30](#_Toc185254383)

[10.4.3	SAEs requiring expedited reporting to the Trial Office	30](#_Toc185254384)

[10.5.	SAE Reporting process	30](#_Toc185254385)

[10.6.	Assessment of causality of an SAE	31](#_Toc185254386)

[10.7.	Assessment of expectedness of an SAE by the CI	32](#_Toc185254387)

[10.8.	Provision of SAE follow-up information	32](#_Toc185254388)

[10.9.	Reporting SAEs to third parties	32](#_Toc185254389)

[11.	DATA HANDLING AND RECORD KEEPING	32](#_Toc185254390)

[11.1.	Source data	33](#_Toc185254391)

[11.2.	Case Report Form (CRF) completion	33](#_Toc185254392)

[11.3.	Participant completed questionnaires	34](#_Toc185254393)

[11.4.	Data management	34](#_Toc185254394)

[11.5.	Self-evident corrections	34](#_Toc185254395)

[11.6.	Data security	34](#_Toc185254396)

[11.7.	Archiving	35](#_Toc185254397)

[12.	END OF TRIAL DEFINITION	35](#_Toc185254398)

[13.	STATISTICAL CONSIDERATIONS	36](#_Toc185254399)

[13.1.	Sample size	36](#_Toc185254400)

[13.2.	Analysis of outcomes	36](#_Toc185254401)

[13.3.	Missing data and sensitivity analyses	36](#_Toc185254402)

[13.4.	Planned final analyses	36](#_Toc185254403)

[14.	ETHICAL CONSIDERATIONS	36](#_Toc185254404)

[15.	CONFIDENTIALITY AND DATA PROTECTION	37](#_Toc185254405)

[16.	FINANCIAL AND OTHER COMPETING INTERESTS	37](#_Toc185254406)

[17.	INSURANCE AND INDEMNITY	37](#_Toc185254407)

[18.	PUBLICATION PLAN	37](#_Toc185254408)

[19.	REFERENCE LIST	38](#_Toc185254409)

# 1. BACKGROUND AND RATIONALE

### 1.1. Background

Painful diabetic neuropathy is highly prevalent, and causes substantial disability and deterioration in quality of life. The mainstay of treatment is pharmacotherapy but is limited by poor efficacy and tolerability [1]. Based on the current trajectory of new drug developments, the emergence and clinical use of more effective compounds is highly unlikely in the next decade [2]. Glycaemic variability (GV) has emerged as a novel, independent risk factor for diabetic neuropathy and correlates with neuropathic pain severity [3,4]. This leads to the hypothesis that reducing GV could contribute to the treatment of painful diabetic neuropathy. However, no intervention study has been conducted to test this hypothesis to date. Hybrid closed-loop (HCL) insulin pumps have proven effective at stabilizing glucose levels and reducing GV. Hence, the main aim of this feasibility/pilot study is to evaluate whether reducing glycaemic variability using hybrid closed-loop insulin pumps can effectively reduce pain intensity in patients with painful diabetic neuropathy. The objectives to meet the aims of this study are:

### 1.2. Justification for design

A randomised crossover trial provides the best opportunity to have adequate power to identify changes in pain intensity whilst accounting for biological variation over the natural time course of the condition. More than 99% of people living with type 1 diabetes in the UK currently have access to continuous glucose monitoring and this would serve as a usual care control. Based on data from our OPTION_DM trial [5], we believe that 12 weeks on therapy should be sufficient to allow optimisation therapy and any change in neuropathic pain scores.

**Advantages of this design:**

- Within-Subject Comparison: Each participant acts as their own control, which can increase the statistical power and reduce variability.
- Efficient Use of Participants: Maximises data collection from each participant, which is advantageous with a small sample size.
- Reduction in Confounding: Helps control for individual differences by comparing outcomes before and after the intervention.
- Use of a crossover design would then allow us to see whether withdrawal of HCL leads to a return in neuropathic pain to baseline. It also allows all participants to access HCL which addresses possible ethical considerations and is important for recruitment in this setting.
- All participants, including those in the control group, eventually receive HCL treatment, which can be crucial when studying potentially beneficial treatments. This helps to address ethical concerns about denying treatment in patients with T1D.

**Risks:**

***Dropout Risk:*** Participants randomised to the control group in the first phase of the study may be dissatisfied and drop out, especially if clinical teams offer them the intervention (e.g., closed loop). Similarly, those randomised to the intervention first may drop out during the second phase when they are required to switch to the control group.

Risk appraisal: Low

Mitigation: We have accounted for a 10% dropout rate and will employ an intention-to-treat analysis to ensure all participants are included in the final analysis, regardless of their completion status. The crossover design is expected to help mitigate dropout risk by allowing participants to experience both interventions. Additionally, participants who withdraw from the study will be asked to provide feedback, which will inform the design of future definitive trials and help refine study procedures.

***Time-Related Changes***: The passage of time can introduce variables that affect outcomes independently of the intervention (e.g., seasonal effects, changes in health status), complicating the interpretation of results.

Risk appraisal: Low

Mitigation: Participants will be on stable neuropathic pain treatment and pain intensity is unlikely to change over the relatively short 6-month study period. The crossover design also mitigates against this.

***Awareness of Group Assignment***: Participants know they are not receiving the active intervention initially, which could affect their reporting of symptoms or adherence to other study protocols. Although assessors can be blinded, participants cannot be fully blinded in a crossover design.

Risk appraisal: High

Mitigation: We are experienced in running clinical pain trials where outcome assessments are subjective and liable to this potential bias. Consistent and supportive communication will be provided to all participants regardless to their group assignment including providing equal monitoring and attention by regular follow up calls/check-ins with all participants, emphasising the uncertainty of treatment outcomes in all study communications, education participants on the importance of accurate/consistent reporting of symptoms (using videos).

### 1.3. Justification for participant population

The eligibility criteria were chosen to facilitate recruitment of the appropriate target population, while excluding patients who may confound study outcomes. The eligibility criteria are therefore designed to identify patients with painful diabetic neuropathy who still have significant pain (mean pain intensity ≥4 on the NRS), consistent with previous trials in the field [5]. The criteria exclude patients with causes of neuropathic pain other than diabetes.

### 1.4. Justification of choice of intervention

Glycaemic variability (GV), characterized by fluctuations in blood glucose levels, is a significant concern in the management of diabetes, particularly in individuals with type 1 diabetes (T1D). Excessive glycaemic fluctuations can contribute to the risk of acute complications such as hypoglycaemia and hyperglycaemia, as well as long-term adverse outcomes, including microvascular and macrovascular complications. Emerging evidence suggests that beyond HbA1c, glycaemic variability may be an independent predictor of diabetes complications, including painful neuropathy. Thus, reducing GV is critical to improving both short-term safety and long-term health outcomes in people with painful diabetic neuropathy.

Challenges in current diabetes management strategies, such as multiple daily injections (MDI) or conventional insulin pump therapy, are often insufficient to maintain stable blood glucose levels, especially in those who face challenges in predicting insulin needs due to variations in diet, physical activity, stress, or illness. Even with intensive management, achieving optimal glycaemic control remains difficult, and individuals are often unable to reduce glycaemic variability effectively. This challenge highlights the need for innovative approaches that offer more precise and responsive glucose control mechanisms.

Hybrid Closed-Loop insulin delivery systems represent a technological advancement that directly addresses the issue of glycaemic variability. These systems integrate CGM with an insulin pump that automatically adjusts basal insulin delivery based on real-time glucose data and predictive algorithms. HCL systems reduce fluctuations by dynamically adjusting insulin dosing to prevent both high and low blood sugar levels. The user still administers bolus insulin for meals, but the automated basal rate adjustments significantly reduce the burden of manual insulin management and help mitigate glycaemic excursions.

1.4.1 HCL vs. regular insulin pumps vs MDI

Hybrid Closed-Loop (HCL) systems are superior to regular insulin pumps or multiple daily injections (MDI) for reducing glycaemic variability due to several key advantages:

Automated Insulin Adjustments: Unlike regular insulin pumps, which deliver a preset basal insulin dose, HCL systems adjust insulin delivery automatically based on continuous glucose readings. This dynamic adjustment helps prevent large glucose swings by fine-tuning insulin delivery in response to real-time glucose fluctuations, reducing both hyperglycaemia and hypoglycaemia.

Predictive Algorithms: HCL systems use advanced algorithms to predict future glucose trends. These algorithms can anticipate rises or drops in blood glucose and adjust insulin delivery pre-emptively to mitigate variability. Regular pumps and MDI rely on manual insulin adjustments, which often lag behind glucose changes.

Continuous Glucose Monitoring (CGM) Integration: HCL systems are integrated with CGM devices, which continuously track glucose levels throughout the day and night. This real-time data enables the system to respond instantly to glucose changes. Regular pumps and MDI, on the other hand, depend on intermittent fingerstick readings, leading to less precise insulin dosing and delayed responses to fluctuations.

Reduced User Burden: With MDI and regular insulin pumps, users must make frequent manual insulin dosing decisions, which can lead to errors in timing or dosage, contributing to glycaemic variability. HCL systems reduce the cognitive burden on the user by automating basal insulin adjustments and providing more consistent glucose control with less need for manual intervention.

Improved Overnight Control: HCL systems are particularly effective in controlling nocturnal glucose levels, a time when variability is harder to manage with manual systems. The ability of HCL to adjust insulin delivery during the night helps prevent both nocturnal hypoglycaemia and morning hyperglycaemia, which are common problems with MDI and traditional pumps.

More Precise Control of Postprandial Glucose: Postprandial glucose spikes are a major contributor to glycaemic variability. While MDI and regular pumps require users to manually calculate and administer bolus insulin doses, HCL systems can help fine-tune insulin delivery around mealtimes by automatically adjusting basal rates, leading to more stable glucose levels after eating.

Lower Risk of Hypoglycaemia: By automatically suspending insulin delivery when glucose levels are predicted to drop too low, HCL systems reduce the risk of hypoglycaemia, which is a common issue with both MDI and regular insulin pumps. This feature helps to maintain glucose within a tighter range, further reducing variability.

In summary, HCL systems provide more precise, responsive, and adaptive insulin delivery compared to regular insulin pumps or MDI, making them more effective in reducing glycaemic variability. Their combination of CGM integration, predictive algorithms, and automated insulin adjustments offers a significant advantage in achieving stable glucose control.

### 1.5. Justification of choice of primary outcome

The primary outcome is the difference in the 7-day mean 24-hour pain score (evaluated at participant level) on an 11-point NRS scale (0 = no pain and 10 = worst pain imaginable) between arms measured at 3 months post randomisation. The 7-day mean 24-hour NRS is considered the gold standard for the assessment of neuropathic pain and has been employed in almost all well-designed neuropathic pain studies over the past 10 years [6-8]. Changes in the 7-day mean NRS were used by the international IMMPACT recommendations and others to determine and validate the clinically important differences in pain intensity [6]. IMMPACT is the Initiative on Methods, Measurement, and Pain Assessment in Clinical Trials, which included a consensus statement from 40 participants from universities, governmental agencies, a patient organisation, and the pharmaceutical industry.

NICE clinical guidance 173 also recommended the use of the 11-point NRS in neuropathic pain trials in the research recommendations section. The European Medicine Agency also recommends the use of the NRS in pain trials [9]. The NRS will be collected daily throughout the study period via text messages or via email (as suggested by the PPI group), but only the average of the 7 days prior to the 3 months-time point will be used for the primary outcome analysis.

We chose the primary outcome to be at 3 months as this is consistent with other trials in the field of painful diabetic neuropathy which were between 6-12 weeks duration [10,11].

# 2. AIMS AND OBJECTIVES

### 2.1. Main trial objectives

2.1.1 Clinical objectives

1. Evaluate the impact of reducing glycaemic variability using HCL insulin pumps on pain intensity compared to control in painful diabetic neuropathy.
2. Evaluate the impact of HCL versus usual care with CGM on glucose metrics (mean glucose, glucose variability, time in range, time in tight range and time below range) in patients with painful neuropathy and their impact on neuropathic pain
3. Evaluate the impact of HCL versus usual care with CGM on other clinically important benefits (quality of life, functional status and psychological well-being)
4. Evaluate the tolerability of HCL in people with painful diabetic neuropathy (assessment of adverse events and serious adverse events)
   2.1.2 Safety objectives

To describe (Serious) Adverse Events data (summarised both at patient level and event level).

2.1.3 Exploratory objectives

1. Neurophysiological assessments (Sudoscan, Vibrosense, DPN-Check and Autonomic Function Tests) will be performed to further understand the underlying mechanisms of painful diabetic neuropathy and the impact of glycaemic variability on these parameters.
2. Sleep parameters (total sleep time, sleep interruption, AHI, Night HR, HRV) and physical activity (active minutes, steps, skin temperature) measured by Withings devices as objective proxy of pain evolution and management.

Feasibility assessments

1. The time needed to collect and analyse data.
2. Screening logs of recruitment and retention strategies: number of eligible participants, different methods of identifying/recruiting patients and willingness of clinicians to recruit participants.
3. Follow-up rates, response rates to questionnaires and adherence/compliance rates.
4. Participant feedback to determine acceptability of the intervention.
5. Exploration of Public Involvement in the research design and its subsequent conduct.
6. Stakeholder (clinician, DSNs) feedback on acceptance of the use of HCL in painful diabetic neuropathy.

# 3. TRIAL DESIGN AND SETTING

PAINLESS is a, two-centre, randomised, cross-over, feasibility study of patients with painful diabetic neuropathy (defined as pain severity of ≥ 4 on 11-point numerical rating scale [NRS] measuring 24-hour pain scores [0 to 10] averaged over the last 7 days).

The primary outcome (24-hour pain NRS averaged over the last 7 days) is measured after each treatment sequence (i.e. at 3 months and 6 months post-randomisation).

Forty patients will be randomised from Sheffield and Leicester that have painful diabetic neuropathy services and the aligned primary care and podiatry services, as well as social media and Diabetes UK local patient groups. Digital promotion will also be used as recruitment strategy to help identify and approach eligible patients. The study delivering the intervention will be placed in the Trusts at each secondary care site.

### 3.1. Assessment of risk

All clinical trials can be considered to involve an element of risk and in accordance with the Sheffield Teaching Hospitals NHS Trust standard operating procedures this trial has been risk assessed to clarify any risks relating uniquely to this trial beyond that associated with usual care. A Risk Assessment has been conducted and concluded that this trial corresponds to the following categorisation: No higher than the risk of standard medical care.

# 4. ELIGIBILITY

### 4.1. Inclusion criteria

- Clinical diagnosis of type 1 diabetes for at least 5 years [commenced on insulin treatment within one year of diagnosis].
- Daily neuropathic pain for at least 3 months.
- Distal symmetrical polyneuropathy confirmed by the Toronto Clinical Neuropathy Score >5 [12].
- 24-hr average pain score > 4 (BPI-MSF) [13].
- Hba1c between 6.5 to 13%.
- No change to dose of neuropathic pain medication within the last month.
- Patients will have a mean total pain intensity of at least 4 on an 11-point numeric rating scale (NRS; with 0 being ‘no pain’ and 10 ‘worst pain imaginable’) during the last week of the Screening Period.
- Willing and able to comply with the study schedule and be available for the treatment duration.
- Able to give written informed consent.

### 4.2. Exclusion criteria

- Non-diabetic neuropathies.
- Other chronic pain disorders more severe that painful diabetic neuropathy.
- History of major psychiatric disorders.
- Pregnancy/breast feeding or planning pregnancy during the course of the study.
- Visual or hearing impairment, which would impede ability to use technological interventions

### 4.3. Co-enrolment

Participants in PAINLESS study can participate in any observational study. Co-enrolment in other non-pain trials can be considered after discussion with members of the Trial Management Group (TMG). The TMG will consist of the study CI and site PIs.

# 5. IDENTIFICATION, RECRUITMENT and CONSENT

### 5.1. Identification

Recruitment will take place from study sites and their aligned podiatry services. A number of approaches will be taken to recruit participants:

1. Patients will be recruited from secondary care diabetes clinics, neuropathy clinics or pain clinics.

2. All currently planned centres have strong links with podiatry services and this link will be used to identify appropriate patients.

3. Study advert will be put in outpatients in the NHS Trusts, primary care, and social media. Diabetes UK local patient groups will publicise the study. When social media is used, we will ensure that the information is clear that this is relevant to patients in certain locations.

All eligible patients will be approached about the trial by a member of their clinical care team who will inform them of the study. This will be in person whilst attending clinic or via invitation letter, email or phone call. Patients will be presented with a Participant Information Sheet (PIS). Patients expressing an interest will be put in touch with the research team, or may contact the research team directly.

Where integrated into the care team, NHS research staff may assist clinicians with caseload screening, use of electronic record searches or research registers/databases of existing patients (e.g. TriNetX) to help identify patients on their caseloads, according to local permissions as appropriate, for any of the methods described above. All patients identified will receive a PIS. This will contain details of the study team to contact for further information. Advertising materials, once developed, will also contain contact details for further information.

The study team will endeavour to recruit patients from a diverse population based on gender at birth, social deprivation and ethnicity. This should be possible considering that painful diabetic neuropathy is strongly associated with socioeconomic disadvantage and is more common in South Asians compared to White Europeans [14,15]. Some of the trial centres are well experienced in recruiting patients from ethnic minorities, such as University of Leicester which has a large South Asian population in the foot clinic. We will also approach the South Asian Health Foundation to publicise the study. Recruitment from primary care and working with the local patient groups will also allow us access to a larger more diverse population than relying on recruitment from secondary care only. We will monitor recruitment and adapt strategies accordingly if needed.

### 5.2. Study consent

It is the responsibility of the Principal Investigator (PI) to obtain written informed consent for each participant prior to any trial related procedures. The process for taking consent can be delegated to members of the local research team working on the trial at the site, all of whom should have undergone Good Clinical Practice (GCP) training. Delegation of this duty will be authorised by the PI and captured on the Site Delegation Log.

The PI or delegate may discuss the study with interested patients during a pre-screening contact (this can be done in clinic or remotely over the phone. The PI or delegate will provide further details of the study, i.e. adequately explain the aim of the trial, the trial interventions, and the anticipated benefits and potential hazards of taking part in the trial and will ensure that the potential participant has the opportunity to ask questions. The PI or delegate will again explain that participation is voluntary and that the participant is free to decide whether to take part in the trial, is not obliged to continue into the main trial, and may withdraw from the trial at any time. Patients will be given sufficient time to consider and, should the participant feel the need to do so, discuss participation with friends and family.If potential participants are interested in taking part they will be invited to attend of a Screening visit at which they will discuss the study with the PI or delegate according to the above as appropriate before being asked to sign and date the latest version of the Informed Consent Form (ICF). A printed copy of the ICF will be given to the participant. A copy of the consent form will be placed in the medical notes. A copy will also be stored electronically in the site-specific section of the database. A copy will be printed for the Investigator Site File (ISF).

If the participant is later randomised into the trial, their trial number will be entered on the copy of the screening ICF maintained in the ISF and the TNO linked against the ICF stored in the database. At the time of screening consent, the participant will be asked to give explicit consent for the signed screening ICF to be stored in the database for internal review and audit purposes.

At each visit the participant’s willingness to continue in the trial will be ascertained and documented in the medical notes. Throughout the trial the participant will have the opportunity to ask questions about it. Any new information that may be relevant to the participant’s continued participation will be provided. Where new information becomes available which may affect the participants’ decision to continue, participants will be given time to consider and if happy to continue will be re-consented. Re-consent will be documented in the medical notes. The participant’s right to withdraw from the trial will remain.

# 6. RANDOMISATION and BLINDING

### 6.1. Randomisation

Once the participant has completed four weeks of the Screening Period with at least a week of daily pain scores (at least 5 out of 7 days must be completed to be considered a valid averaged score) and the participant fulfils inclusion criteria, their eligibility will be assessed. The PI or delegate will reconfirm the participant is still willing to take part in the study. If the participant confirms they are happy to enrol into the trial, they will be randomised (see section 6.4).

All eligible participants will be randomised. Randomisation will be performed using a secure online randomisation programme [https://www.sealedenvelope.com/simple-randomiser/v1/lists] thereby ensuring allocation concealment. Unique log-in usernames and passwords will be provided to those who have been delegated the role of randomising participants into the trial as detailed on the PAINLESS study Site Delegation Log. These unique log-in details must not be shared with other staff and in no circumstances should staff at sites access the system using another person’s login details. The online system will be available 24 hours a day, 7 days a week, apart from short periods of scheduled maintenance. In the rare case of a technical failure within the online randomisation system an emergency paper back up can be used.

6.1.1 Randomisation method

Participants will be randomised at the level of the individual in a 1:1 ratio to either Treatment Sequence A (HCL+CGM followed by CGM only) or Treatment Sequence B (CGM only followed by HCL+CGM). The randomisation will use a stratification algorithm to ensure balance in the treatment allocation taking into account age (18-39, 40-59, 60+), and sex at birth (male/female).

### 6.2. Blinding

Given the nature of the HCL intervention and the use of a visible device, it is not possible to blind participants to their assigned treatment arm. To mitigate potential bias from research staff, pain surveys are completed solely by the participants, with other secondary outcome assessments such as neurophysiological assessments and haemoglobin A1c yielding objective numerical data, which are not subject to assessor.

### 6.3. Informing the participant’s GP and other parties

If the participant has agreed, the participant’s GP should be notified that they are in the PAINLESS study, using the PAINLESS Study GP Letter.

# 7. TRIAL INTERVENTION

### 7.1. Trial intervention and dosing schedule

At the baseline visit (v2), participants will be randomised to:

- Treatment Sequence A: Hybrid closed loop with Tandem T slim pump and Dexcom G7 sensors using Control iQ algorithm

OR

- Treatment Sequence B: CGM alone with Dexcom G7

It is expected that HCL treatment start will be performed in groups (2-4 participants per group) wherever possible whilst still ensuring that treatment is initiated within 3 months of randomisation to avoid the need for baseline assessments to be repeated.

### 7.2. Interactions or contraindications

7.2.1 Concomitant medication(s)/intervention(s)

Regular pain medication should continue as per routine care during trial participation.

7.2.2 Prohibited medication(s)/intervention(s)

There are no prohibited medications or interventions in the PAINLESS study.

### 7.3. Post-trial care

There is no provision for post-trial care. Participants will return to their usual care providers. At the end of the study, devices will need to be returned. However, if the participant has gained benefit from the devices and are eligible on NHS as per NICE guidelines, we will liaise with the local team to facilitate continuation on equivalent technology where possible and ask them to take over ordering of supplies.

# 8. OUTCOME MEASURES

### 8.1. Main trial outcomes

All the outcomes listed in this section are in line with IMMPACT consensus recommendations for interpreting the clinical importance of treatment outcomes in clinical trials of the efficacy and effectiveness of chronic pain treatments [6]. IMMPACT highlights 4 core domains:

1. Pain intensity, assessed by a 0 to 10 NRS
2. Physical functioning
3. Emotional functioning
4. Participant ratings of overall improvement, assessed by the PGIC and Clinician Global Impression of Change (CGIC) with a 7-point Likert scale.

### 8.2. Primary outcome

Change in pain as measured by the 7-day average 24hour pain numeric rating scale of 0-10 (0=no pain and 10= worse pain imaginable) from baseline to the final follow up week (3 months post-randomisation).

### 8.3. Secondary Outcome

8.3.1 Clinical

1. Treatment success (measured as a 30% reduction in 7-day average 24-hour pain scores) at end of the study.
2. Treatment success (measured as a 50% reduction in 7-day average 24-hour pain scores) at end of the study.
3. Area Under the Curve (AUC) for the daily NRS pain scores over the study period (from baseline to the study).
4. Brief Pain Inventory-MSF (BPI-MSF) as a measure of pain interference with function total score at end of the study.
5. Beck Depression Inventory as a measure of depression at end of the study.
6. Neuropathic Pain Symptom Inventory (NPSI) as a measure of pain intensity at end of the study.
7. Patient and Clinician Global Impression of Change at end of the study.
   8.3.2 Safety
1. Frequency and proportion of Adverse Events.
2. Frequency of Serious Adverse Events.

### 8.4. Exploratory Endpoint

1.	Neurophysiological assessments (autonomic function tests, Sudoscan, Vibrosense and DPN-Check) will be performed to further understand the underlying mechanisms of painful diabetic neuropathy and the impact of glycaemic variability on these parameters.

2.	Sleep parameters (total sleep time, sleep interruption, apnea-hypopnea index, night heart rate, heart rate variability) and physical activity (active minutes, steps, skin temperature) measured by Withings devices as objective proxy of pain evolution and management.

# 9. TRIAL PROCEDURES

The intervention will be delivered by a research nurse or a Clinical Research Fellow.

For trial assessments please see Table 1. The trial visits and data collected are summarised below:

Visit 1 (Week 0, Screening Visit): Written informed consent and screening. Participants will provide written informed consent. They will have the opportunity to ask any questions and to see the devices that they will be randomised to. We will give them links to the diabetes technology network website where they can watch further videos around hybrid closed loop. Once consent is obtained, we will screen them for inclusion and exclusion criteria and collect baseline information that will include:

1. Current treatment and medication
2. Medical history, including detailed history of their neuropathic pain
3. Clinical examination including the DN-4 questionnaire (Screening neuropathic pain questionnaire), Toronto Clinical Neuropathy Score (structured clinical neurological assessment)
4. Questionnaires: Neuropathic Pain Symptom Inventory (NPSI), Brief Pain Inventory-Modified Short Form (BPI-MSF) [13]. Beck’s Depression Inventory (BDI) [16]. SF-36 [17] and EuroQol-5D-5L [18], type 1 diabetes distress score (T1-DDS), Hypoglycaemia Awareness Questionnaire (HypoAQ) [19], Hypo confidence score [20].
5. Neurophysiological assessments of small (autonomic function tests, Sudoscan, Impeto Medical ltd) and large (DPN-Check, NeuroMetrix Inc) fibres will be performed.
6. Safety laboratory blood samples (glycosylated haemoglobin A1c) will be performed if not available in the previous 3 months.

- Participants will be provided with Dexcom G7 sensors to ensure that all the CGM data collected with the same device
- We will collect 4 weeks of baseline glucose data along with pain diaries. Each morning patients will be instructed to assess total pain experienced over the preceding 24-hours, rated once daily, using an 11-point NRS. (with 0 being ‘no pain’ and 10 ‘worse possible pain’), record the amount of rescue medication used. From the daily pain scores, a mean for each week will be determined and used in subsequent analysis.
- Baseline physical activity and sleep analysis will be performed using a Withings Sleep Analyser and Withings’ ScanWatch2 or Fitbit Inspire2 fitness/activity tracker. Participants will continue using these devices till the end of the study.

Visit 2 (Week 4): At the end of the screening period. We will download CGM data to ensure compliance with CGM. They will also complete insulin dose diaries so we will have measure of the total daily dose. Screening period pain diaries will be checked to confirm patients’ eligibility (mean NRS pain score ≥4) and stored. Review of concomitant medications performed. Study eligibility will be verified before participants are randomised to one of 2 treatment sequences (A or B) using an online randomisation program [https://www.sealedenvelope.com/simple-randomiser/v1/lists].

Study eligibility will be assessed remotely and eligible participants will be randomised prior to Visit 2. Those randomised to HCL (Treatment Sequence A) will be batched wherever possible whilst still ensuring that treatment is initiated within 3 months of randomisation to avoid the need for baseline assessments to be repeated. They will be seen face-to-face to commence on HCL pumps using tandem T slim insulin pumps with Dexcom G7 sensors and the control IQ algorithm as per national guidelines [21] in groups (maximum 4). Participants randomised to Treatment Sequence B will continue on usual care and next assessed at Visit 3B.

**Treatment Sequence A**: HCL and Dexcom G7 CGM

Visit 3A (Week 4+3 days)

Three days after the HCL start, we will contact participants by telephone to ensure that the HCL is working okay and to troubleshoot any issues. We will also ensure that they managed to do a set change safely and looking at glucose downloaded he will make any modifications to therapy is required.

Visit 4A (Week 6)

At this face-to-face visit HCL pump settings will be optimised using national guidance. Daily pain diaries will be collected and new ones provided.

Visit 5A (Week 8)

This visit may be conducted virtually or face-to-face. We will review glucose data to optimise HCL pump use to minimise glucose variability.

Visit 6A (Week 12)

This visit may be conducted virtually or face-to-face. We will review glucose data and try to optimise the HCL use to minimise glucose variability.

Visit 7A (Week 16)

Pain diaries will be collected and stored. Assessments of compliance and adverse events will be conducted. The following assessments will be performed:

1.	Download of HCL data.

2.	Lab sample for HbA1c.

3.	Neuropathic pain assessment: Pain diaries for the last 4 weeks of the intervention period and BPI-MSF.

4.	Psychological and quality of life assessments: Mood assessed by BDI. Health status assessed by SF-36, EuroQoL-5D-5L will be completed.

5.	Neurophysiological assessments of small (Sudoscan, Impeto Medical ltd) and large (DPN-Check, NeuroMetrix Inc) nerves, vibration (VibroSense Meter II, Vibrosense) and autonomic nerve function (Vagus, Medicus Engineering ApS) will be performed.

Participants will then cross-over into Treatment Sequence B for the second half of the study.

**Treatment Sequence B**: Dexcom G7 CGM only

Visit 3B (Week 6)

Participants will be contacted by telephone to review glucose data in order to optimise insulin therapy.

Visit 4B (Week 8)

During this virtual or face-to-face visit, we will review glucose data to optimise insulin therapy.

Visit 5B (Week 12)

During this virtual or face-to-face visit, we will review glucose data to optimise insulin therapy. Participants will be provided with pain diaries to complete prior to the next visit.

Visit 6B (Week 16):

Pain diaries will be collected and stored. Assessments of compliance and adverse events will be conducted. The following assessments will be performed:

1.	Download of CGM data

2.	Lab sample for HbA1c

3.	Neuropathic pain assessment: Pain diaries for the last 4 weeks of the intervention period, and BPI-MSF.

4.	Psychological and quality of life assessments: Mood assessed by BDI. Health status assessed by SF-36, EuroQoL-5D-5L will be completed.

5.	Neurophysiological assessments of small and large nerves, vibration and autonomic nerve function will be performed.

Participants will then cross-over into Group A for the second half of the study.

Upon completion of the study participants and study personnel will complete the relevant Global Impression of Change questionnaire.

### 9.1. Schedule of assessments

Table 1a: Schedule of Assessments

| Assessment | V1 | V2 | V3A/B | V4A/B | V5A/B | V6A | V6B/7A |
| --- | --- | --- | --- | --- | --- | --- | --- |
| Informed consent | X |  |  |  |  |  |  |
| Eligibility checked | X | X |  |  |  |  | X |
| Questionnaires | X |  |  |  |  |  | X |
| Blood tests | X |  |  |  |  |  | X |
| Neurophysiological tests | X |  |  |  |  |  | X |
| CGM download | X | X |  |  |  |  |  |
| Randomisation |  | X |  |  |  |  |  |
| HCL pump start |  | X |  |  |  |  |  |
| CGM HCL/Insulin optimisation |  |  | X | X | X | X |  |

Table 1b: Schedule of Assessments – questionnaire details

| Assessment | Screening (V1) | Baseline (V2) | End of treatment V7A/6B) |
| --- | --- | --- | --- |
| Informed consent | X |  |  |
| Eligibility checked | X | X |  |
| NRS* | X | X | X |
| TCNS | X |  | X |
| DN-4 | X |  |  |
| NPSI | X |  | X |
| Pain medications | X | X | X |
| Patient GIC |  |  | X |
| Clinician GIC |  |  | X |
| BDI | X |  | X |
| EQ5D-5L | X |  | X |
| SF-36 | X |  | X |
| TIDDS | X |  | X |
| BPI-MSF | X |  | X |
| HypoA-Q | X |  | X |
| Hypo Confidence Score | X |  | X |
| HbA1c | X |  | X |
| Med history and examination | X |  |  |
| Neurophysiological tests | X |  | X |
| AE review** |  |  | X |

*NRS*: collected daily via pain diaries and the average of the last 7 days will be calculated at each time point. **AE will be documented at each study visit.*

### 9.2. Withdrawal and changes in levels of participation

Informed consent is defined as the process of learning the key facts about a clinical trial before deciding whether or not to participate. It is a continuous and dynamic process and participants should be asked about their ongoing willingness to continue participation at all visits. Participants should be aware from the beginning that they can freely withdraw (cease to participate) from the trial at any time. A participant may wish to cease to participate in a particular aspect of the trial.

Participants found to be ineligible post-randomisation should be followed up according to all trial processes and will still have their data analysed as per an ‘intention to treat’ approach unless they explicitly change their level of participation.

The changes in levels of participation within the trial are categorised in the following ways:

No trial intervention: The participant would no longer like to receive the trial intervention but is willing to be followed up in accordance with the schedule of assessments and if applicable using any central UK NHS bodies for long-term outcomes (i.e. the participant has agreed that data can be collected and used in the trial analysis).

No trial related follow-up: The participant does not wish to attend trial visits in accordance with the schedule of assessments but is willing to be followed up at standard clinic visits and if applicable using any central UK NHS bodies for long-term outcomes (i.e. the participant has agreed that data can be collected at standard clinic visits and used in the trial analysis, including data collected as part of long-term outcomes).

No further data collection: The participant is not willing to be followed up in any way for the purposes of the trial AND does not wish for any further data to be collected (i.e. only data collected prior to any changes of levels in participation can be used in the trial analysis).

The details of changes of levels in participation within trial (date, reason and category of status change) should be clearly documented in the source documents.

# 10. ADVERSE EVENTS REPORTING

### 10.1. Definitions

Table 6: Adverse event reporting definitions

| Severity Definitions | Mild  Moderate  Severe | Awareness of signs or symptoms that do not interfere with the participant’s usual activity or are transient and resolved without treatment and with no sequelae.  A sign or symptom, which interferes with the participant’s usual activity.  Incapacity with inability to do work or perform usual activities. |
| --- | --- | --- |
| Adverse Event | AE | Any untoward medical occurrence in a participant participating in the trial which does not necessarily have a causal relationship with the intervention received. |
| Related Event | RE | An event which resulted from the administration of any of the research procedures. |
| Serious Adverse Event | SAE | An untoward occurrence that:  Results in death  Is life-threatening*  Requires hospitalisation or prolongation of existing hospitalisation  Results in persistent or significant disability or incapacity  Consists of a congenital anomaly/ birth defect  Or is otherwise considered medically significant by the Investigator** |
| Unexpected Event | UE | The type of event that is not listed in the protocol as an expected occurrence. |
| Related and Unexpected Serious Adverse Event | N/A | A SAE that meets both the definition of a Related and Unexpected Event. |

* The term life-threatening is defined as diseases or conditions where the likelihood of death is high unless the course of the disease is interrupted.

** Medical events that may not be immediately life-threatening or result in death or hospitalisation but may jeopardise the participant or may require intervention to prevent one of the other outcomes listed in the definitions above.

### 10.2. Adverse event recording – general

The recording and reporting of Adverse Events (AEs) will be in accordance with the UK Policy Framework for Health and Social Care Research, the Principles of GCP as set out in the UK Statutory Instrument (2004/1031; and subsequent amendments) and the requirements of the Health Research Authority (HRA). Definitions for adverse event reporting are listed in Table 6: Adverse event reporting definitions in Section 10.1.

It is routine practice to record AEs in the participant's medical notes and it is also recommended that this includes the documentation of the assessment of severity and seriousness and also of causality (relatedness) in relation to the intervention(s) in accordance with the protocol.

### 10.3. AE reporting in PAINLESS STUDY

The reporting period for AEs in PAINLESS Study will be from the start of treatment until the end of trial follow-up.

The safety profile for this trial population and interventions are well characterised so a strategy of targeted reporting of AEs will not affect the safety of participants. Only those AEs detailed below will be reported:

•	Serious Adverse Events (SAEs) (reported on trial specific SAE CRF).

•	AEs possibly or definitely related to trial treatment, occurring between the start of trial treatment and 7 days after the end of the trial treatment period (reported on trial specific AE log).

### 10.4. SAE reporting in PAINLESS STUDY

For all SAEs, the PI or delegate must do one of the following:

1. Record safety reporting-exempt SAEs in the medical notes but not report them to the Trial Office on an SAE form as per Section 10.4.1.
2. Report SAEs to the trial office in a non-expedited manner. This can only be done for the pre-defined subset of SAEs as per Section 10.4.2.
3. Report SAEs to the trial office in an expedited manner. All SAEs not covered by the above 2 categories must be reported within 24 hours of the site research team becoming aware of the event.

Note: when an SAE occurs at the same hospital at which the participant is receiving trial intervention or is being followed up for trial purposes, processes must be in place to make the trial team at the hospital aware of any SAEs, regardless of which department first becomes aware of the event, in an expedited manner.

Note: For purposes of adverse event reporting, the Trial Office is defined as the Chief Investigator (or in their absence, a nominated deputy). Reporting will be conducted via an email directed at [d.selvarajah@sheffield.ac.uk](mailto:d.selvarajah@sheffield.ac.uk). If he is not present, an automatic reply will direct the sender to the nominated deputy.

10.4.1 SAEs not requiring reporting to the Trial Office

At whatever time they occur during an individual’s participation, during the AE reporting period, the following are not considered to be critical to evaluations of the safety of the trial:

•	Pre-planned hospitalisation

•	Hospital admissions lasting less than 24 hours

All events which meet the definition of serious must be recorded in the participant notes, including the causality and severity, throughout the participant’s time on trial, including follow-up, but for trial purposes these events do not require reporting on the SAE Form. Such events are “safety reporting exempt”.

10.4.2 SAEs requiring non-expedited reporting to the Trial Office

Where the safety profile is well established, the known potential adverse effects of the intervention (or the participant’s underlying condition) may be known. Such events are protocol-defined as “expected” (see Section 10.7 Assessment of expectedness of an SAE by the CI).

Such events should still be recorded by the trial team in the participant’s notes and reported to the Trial Office on the trial specific SAE form but do not require expedited reporting (immediately on the site becoming aware of the event) since the assessment of expectedness for the specified events has been pre-defined. It is expected that these SAEs are reported within 7 days of the site becoming aware of the event. These include:

•	Hospitalisations related to DM or other pre-existing conditions and not related to the trial intervention

SAEs requiring expedited reporting to the Trial Office

All SAEs not listed in Sections 10.4.1 and 10.7 must be reported to the Trial Office on the trial specific SAE form within 24 hours of the site research team becoming aware of the event.

### 10.5. SAE Reporting process

On becoming aware that a participant has experienced an SAE which requires reporting on an SAE form, the PI or delegate should report the SAE to their own Trust in accordance with local practice and to the Trial Office.

To report an SAE to the Trial Office, the PI or delegate must complete, date and sign the trial specific SAE form. The completed form together with any other relevant, appropriately anonymised, data should be submitted to the Trial Office using the information below in accordance with the timelines given in Section 10.4.2 and 10.4.3.

To report an SAE, submit the SAE Form to relevant parties that will be detailed on the form.

Where an SAE Form has been completed by someone other than the PI initially, the original SAE form must be countersigned by the PI to confirm agreement with the causality and severity assessments.

On receipt of an SAE form, the Trial Office will allocate each SAE a unique reference number and notify the site via email to the site as proof of receipt. The site and the Trial Office should ensure that the SAE reference number is quoted on all correspondence and follow-up reports regarding the SAE and filed with the SAE in the ISF.

If the site has not received confirmation of receipt of the SAE or if the SAE has not been assigned a unique SAE identification number within 1 working day of reporting, the site should contact the Trial Office.

### 10.6. Assessment of causality of an SAE

When completing the SAE form, the PI (or, throughout this section, a medically qualified delegate) will be asked to define the nature of the seriousness and causality (relatedness; see Table 2: Categories of causality) of the event.

In defining the causality, the PI must consider if any concomitant events or medications may have contributed to the event and, where this is so, these events or medications should be reported on the SAE form. It is not necessary to report concomitant events or medications which did not contribute to the event.

As per Table 2, all events considered to be ‘possibly’, ‘probably’, or ‘definitely’ related to the intervention will be reported by the Trial Office as ‘related’; all events considered at site to be ‘unlikely’ or ‘unrelated’ to the intervention will be reported by the Trial Office as ‘unrelated’. The same categorisation should be used when describing AEs and protocol-exempt SAEs in the source data.

Table 2: Categories of causality

| Category | Definition | Causality |
| --- | --- | --- |
| Definitely | There is clear evidence to suggest a causal relationship, and other possible contributing factors can be ruled out. | Related |
| Probably | There is evidence to suggest a causal relationship, and the influence of other factors is unlikely. |  |
| Possibly | There is some evidence to suggest a causal relationship. However, the influence of other factors may have contributed to the event (e.g. the participant’s clinical condition, other concomitant events or medication) |  |
| Unlikely | There is little evidence to suggest there is a causal relationship. There is another reasonable explanation for the event (e.g. the participant’s clinical condition, other concomitant events or medication). | Unrelated |
| Not related | There is no evidence of any causal relationship. |  |

On receipt of an SAE Form, the Trial Office will forward it, with the unique reference number, to the Chief Investigator (CI) or delegate who will independently* review the causality of the SAE. An SAE judged by the PI or CI or delegate to have a reasonable causal relationship (“Related” as per Table 7: Categories of causality) with the intervention will be regarded as a related SAE. The severity and causality assessment given by the PI will not be downgraded by the CI or delegate. If the CI or delegate disagrees with the PI’s causality assessment, the opinion of both parties will be documented, and where the event requires further reporting, the opinion will be provided with the report.

*Where the CI is also the reporting PI an independent clinical causality review will be performed.

### 10.7. Assessment of expectedness of an SAE by the CI

The CI or delegate will assess related SAEs for expectedness with reference to the criteria in Table 3.

Table 3: Categories of expectedness

| Category | Definition |
| --- | --- |
| Expected | An adverse event that is consistent with known information about the trial related procedures, trial intervention (as described in the manufacturer’s guidance) or pre-existing conditions of the participant (including DM). |
| Unexpected | An adverse event that is not consistent with known information about the trial related procedures, trial intervention, or pre-existing conditions of the participant. |

If the event is unexpected (i.e. it is not defined in the protocol as an expected event) it will be classified as a related and unexpected SAE.

The CI will undertake review of all SAEs and may request further information from the clinical team at site for any given event to assist in this.

### 10.8. Provision of SAE follow-up information

Following reporting of an SAE for a participant, the participant should be followed up until resolution or stabilisation of the event. Follow-up information should be provided using the SAE reference number provided by the Trial Office. Once the SAE has been resolved, all critical follow-up information has been received and the paperwork is complete, a copy of the final version of the completed SAE form must be submitted to the Trial Office and a copy printed to be stored in the ISF.

### 10.9. Reporting SAEs to third parties

The Trial Management Group may review any SAEs at their meetings.

The Trial Office will report all events categorised as Unexpected and Related SAEs to the REC and Sponsor within 15 days of being notified.

Any Dexcom G7-related serious adverse events will be reported to Dexcom by the trial office within 24 hours of notification of the SAE. Documents associated with the SAE, for example, lab reports, death summaries, and operative reports, will be subsequently shared with Dexcom, redacted of identifiers. Any non-serious device related adverse events will be shared with Dexcom within one week of notification of the AE.

All ‘Reportable Device Issues’ related to the Tandem T:Slim X2 insulin pump will be reported to Tandem within one week of the local site/ trial office being made aware. Any written, electronic or oral communication received shall be considered a ‘Reportable Device Issue’ if there is an allegation of deficiencies related to the identity, quality, durability, reliability, safety, effectiveness or performance of the Study Device or the documentation, regardless of whether the Study Device is returned by the user. AEs related to the Tandem T:Slim X2 insulin pump will be treated as a ‘Reportable Device Issue’. It might relate to either the Study Device or related documentation. In case of a reportable device issue, the following information will be collected:

(1) the date of the Product Complaint,
(2) the Study Device product name and serial or lot number,
(3) the date sequence of events surrounding the complaint, including, where applicable, the date the user of the Study Device became aware of the alleged deficiency in the Study Device or documentation, and
(4) the clinical impact, if any, on the user.

All SAEs, deaths and pregnancies, whether related or unrelated to the T:Slim X2 device, will be reported promptly to Tandem.

An Unanticipated Adverse Device Effect (UADE), as defined as an adverse event related to the T:Slim X2 device which is unexpected (as defined above), will be reported to Tandem within 24 hours of the trial office being made aware of the event.

Details of all Unexpected and Related SAEs and any other safety issue which arises during the course of the trial will be reported to the PIs. A copy of any such correspondence should be filed in the ISF and Trial Master File (TMF).

# 11.  DATA HANDLING AND RECORD KEEPING

### 11.1. Source data

Source data is defined as all information in original records and certified copies of original records of clinical findings, observations, or other activities in a clinical trial necessary for the reconstruction and evaluation of the trial. In order to allow for the accurate reconstruction of the trial and clinical management of participants, source data will be accessible and maintained.

In the PAINLESS Study, some data variables may be entered directly onto the CRF; these are clearly identified and detailed in Table 9: Source data in PAINLESS Study.

Table 9: Source data in PAINLESS STUDY

| Data | Source |
| --- | --- |
| Participant Reported Outcomes | The original completed physical copies are the source and will be held at study sites. |
| Pain Numerical Rating Scale | The participant’s responses will be recorded in Pain Diaries and will be held at study sites. |
| Baseline and end of study CRFs | Data is entered directly onto paper CRFs which is the source and held at study sites. |
| Clinical event data | The original clinical annotation is the source document. This may be found on clinical correspondence, or electronic or paper participant records. Clinical events reported by the participant, either in or out of clinic (e.g. phone calls), must be documented in the source documents. |
| Recruitment | The randomisation CRF is the source. It is held at study sites. |
| Change of status | The change of status form is the source data and will be held at study sites. This form will be completed by site research staff. |

### 11.2. Case Report Form (CRF) completion

The CRFs will include (but will NOT be limited to) the following Forms (see Table 10).

Table 10: Case report forms in PAINLESS STUDY

| Form Name | Schedule for submission |
| --- | --- |
| Consent Form | At the point of completing eligibility screening |
| Pain diary | Should be completed daily. Sites will enter scores at the end of the screening phase ( and in monthly batches during follow up. |
| Informed Consent Form | At the point of consent |
| Participant Contact Details Form | At the point of consent |
| Baseline CRF | As soon as possible after consent |
| Randomisation CRF | At the point of randomisation |
| End of treatment CRF | As close to the end of treatment date as possible |
| Change of Status Form | At the point of change of status, withdrawal or death |
| SAE reporting Form | At the point of being aware of an SAE |

A CRF should be completed for each individual participant. Data should be submitted in a timely manner i.e. within four weeks of submission schedule.

In all cases it remains the responsibility of the PI to ensure that the CRF has been completed correctly and that the data are accurate. This will be evidenced by the electronic sign off of the PI or delegate. The Site Delegation Log will identify all those personnel with responsibilities for data collection.

The delegated staff completing the CRF should ensure the accuracy, completeness and timeliness of the data reported. This will be evidenced by the electronic sign off of the CRF.

Data reported on each CRF will be consistent with the source data and any discrepancies will be explained. All missing and ambiguous data will be queried. Staff delegated to complete CRFs will be trained to adhere to PAINLESS Study CRF completion guidelines.

The following guidance applies to data and partial data:

•	Time format – all times should be in accordance with the 24hr clock.

•	Rounding conventions – rounding should be to the nearest whole number: If the number you are rounding is followed by 5, 6, 7, 8, or 9, round the number up. Example: 3.8 rounded to the nearest whole number is 4. If the number you are rounding is followed by 1, 2, 3 or 4, round the number down. Example: 3.4 rounded to the nearest whole number is 3.

•	Trial-specific interpretation of data fields – where guidance is needed additional information will be supplied.

•	Entry requirements for concomitant medications (generic or brand names) – generic names should be used where possible.

•	Missing/incomplete data – should be clearly indicated – all blank fields will be queried by the Trial Office.

•	Protocol and GCP non-compliances should be reported to the Trial Office on discovery.

### 11.3. Participant completed questionnaires

The NRS pain diary will be completed daily, with telephone reminders sent to complete by return text. Other participant completed questionnaires will be completed on paper in at study visits. The participant will complete under the supervision of a local researcher, who should ensure completeness.

Questions relating to suicidal thoughts are asked in some of the participant completed questionnaires. Should the patient answer yes to these the patient will be flagged as a risk. It will be the sites responsibility to act accordingly and follow this up with the patient in line with their local policy.

### 11.4. Data management

Processes will be employed to facilitate the accuracy and completeness of the data included in the final report. These processes will be detailed in the trial specific Data Management Plan and include the processes of data entry and data queries.

### 11.5. Self-evident corrections

No self-evident corrections will be permitted in the PAINLESS Study.

### 11.6. Data security

Sheffield Teaching Hospitals NHS Trust has policies in place which are designed to protect the security, accuracy, integrity and confidentiality of Personal Data. The trial will be registered with the Data Protection Officer and will hold data in accordance with the Data Protection Act (2018 and subsequent amendments). The Trial Office has arrangements in place for the secure storage and processing of the trial data which comply with Trust policies.

The Trial Database System incorporates the following security countermeasures:

Physical security measures: restricted access to the building, supervised onsite repairs and storages of back-up tapes/disks are stored in a fire-proof safe.

Logical measures for access control and privilege management: including restricted accessibility, access-controlled servers, separate controls of non-identifiable data.

Network security measures: including site firewalls, antivirus software and separate secure network protected hosting.

System management: the system will be developed by the Programming Team at the Trial Office and will be implemented and maintained by the Programming Team.

System design: the system will comprise of a database and a data entry application with firewalls, restricted access, encryption and role-based security controls.

Operational processes: the data will be processed and stored within Sheffield Teaching Hospitals NHS Trust.

System audit: The system will benefit from the following internal/external audit arrangements:

- Internal audit of the system
- Periodic IT risk assessment

Data Protection Registration: Sheffield Teaching Hospital’s Data Protection Registration number is Z8751481.

Participants using the Dexcom G7 will consent to upload their data to the Dexcom Clarity app which is the clinically used and approved app that is required for the patient and the clinican to be able to view their glucose data.

Participants using the Tandem Control iQ systems will be asked to consent to upload their data to the Glooko or tandem t-connect app which are the clinically relevant apps required for patients and clinicians to view their data.

### 11.7. Archiving

It is the responsibility of the PI to ensure all essential trial documentation and source documents (e.g. signed ICFs, Investigator Site Files, participants’ hospital notes, copies of paper CRFs) at their site are securely retained for the contractual period (at least 15 years). Archiving will be authorised by the Sponsor following submission of the end of trial report. No documents should be destroyed without prior approval from the Sponsor’s Director or their delegate.

The TMF will be stored at Sheffield Teaching Hospitals NHS Trust for at least 3 years after the end of the trial. Long-term offsite data archiving facilities will be considered for storage after this time; data will be stored securely and confidentially for at least 15 years. The Trust has standard processes for both hard copy and computer database legacy archiving.

# 12.  END OF TRIAL DEFINITION

The end of trial will be the date of the last data capture including confirmation of resolution of all DCFs. This will allow sufficient time for the completion of protocol procedures, data collection and input and data cleaning. The Trial Office will notify the REC and the Sponsor within 90 days of the end of trial. The Trial Office will provide the REC and the Sponsor with a summary of the clinical trial report within 12 months of the end of trial.

# 13.  STATISTICAL CONSIDERATIONS

### 13.1. Sample size

As this is a feasibility study there is no formal hypothesis testing. NIHR guidelines suggest a minimum sample size of 12 per group is sufficient to help in the design of future studies [22]. Database lock will take place once all data entries have been checked and sufficient verification processes are completed.

Proposed sample size for a definitive study: One-point NRS change is considered clinically significant [6]. Hence, using 1.0 NRS change, within subject SD of 1.8, two-sided significance level of 0.05, and 90% power, assuming 10% attrition, we require 136 subjects (68 per group). This sample size will ensure adequate power to detect the hypothesised 1-point change in pain scores between the study arms. To account for possible dropouts (up to 20%) we will randomise between 40-49 participants to achieve a sample size of 40 participants. A range is provided as we will closely monitor dropout rates and strategically randomise participants to ensure we minimise the overall number of participants required.

### 13.2. Analysis of outcomes

The primary analysis will be conducted using an intention-to-treat approach. Mixed-effects models will be used to assess the impact of the intervention on pain intensity, adjusting for potential confounders. Subgroup analyses will be used to relate patient phenotype to treatment response.

### 13.3. Missing data and sensitivity analyses

Every attempt will be made to collect full follow-up data on all study participants; it is thus anticipated that missing data will be minimal. Participants with missing primary outcome data will not be included in the primary analysis in the first instance (NRS pain score will be considered as missing if fewer than 5 days recorded). This presents a risk of bias, and sensitivity analyses will be undertaken to assess the possible impact of the risk. In brief, this will include a multiple imputation approach. Further sensitivity analysis will include a per protocol analysis.

### 13.4. Planned final analyses

The primary analysis for the trial will occur once all participants have completed the 6-month assessment and corresponding outcome data has been entered onto the trial database and validated as being ready for analysis. This analysis will include data items up to and including the 6-month assessment and no further.

# 14.  ETHICAL CONSIDERATIONS

The trial will be conducted in accordance with the UK Policy Framework for Health and Social Care Research and applicable UK Acts of Parliament and Statutory Instruments (and relevant subsequent amendments), which include Data Protection Act 2018; and the Principles of GCP. The protocol will be submitted to and given a favourable opinion by the REC prior to the start of the trial.

Before any participants are enrolled into the trial, the PI at each site is required to obtain the necessary local approval.

It is the responsibility of the PI to ensure that all subsequent amendments gain the necessary local approval. This does not affect the individual clinicians’ responsibility to take immediate action if thought necessary to protect the health and interest of individual participants.

# 15.  CONFIDENTIALITY AND DATA PROTECTION

Personal data and sensitive personal data recorded on all documents will be regarded as strictly confidential and will be handled and stored in accordance with the Data Protection Act 2018 (and subsequent amendments).

Participants will only be identified by their unique trial identification number and initials on CRFs (except the ICF and Randomisation form) and on any correspondence with the Trial Office. For all participants full name, full date of birth, gender at birth and NHS number will be collected on the Randomisation Form. The participant’s full name will also be collected on the participant consent forms in addition to their email address and/or mobile number.

The PI must maintain documents not for submission to the Trial Office (e.g. Participant Recruitment and Identification Logs) in strict confidence. In the case of specific issues and/or queries from the regulatory authorities, it will be necessary to have access to the complete trial records. Representatives of the PAINLESS Study team and sponsor may be required to have access to participants’ notes for quality assurance purposes, but participants should be reassured that their confidentiality will be respected at all times. The Trial Office will maintain the confidentiality of all participant data and will not disclose information by which participants may be identified to any third party.

# 16.  FINANCIAL AND OTHER COMPETING INTERESTS

There are no financial or other competing interests related to the results of this trial. Authors are similarly required to provide declarations at the time of submission to publishers.

# 17.  INSURANCE AND INDEMNITY

Sheffield Teaching Hospitals NHS Trust has in place Clinical Trials indemnity coverage for this trial which provides cover harm which comes about through the Trust’s, or its staff’s, negligence in relation to the design or management of the trial and provide cover for non-negligent harm to participants.

With respect to the conduct of the trial at Site and other clinical care of the patient, responsibility for the care of the patients remains with the NHS organisation responsible for the Clinical Site and is therefore indemnified through the NHS Resolution.

# 18.  PUBLICATION PLAN

On completion of the trial, the data will be analysed, and a Final Study Report will be prepared. Results of this trial will be submitted for publication in a peer reviewed journal. Manuscripts will be prepared by the writing group as defined in the trial publication plan. Any secondary publications and presentations prepared by Investigators must be reviewed and approved by the TMG. Manuscripts must be submitted to the TMG in a timely fashion and in advance of being submitted for publication, to allow time for review and resolution of any outstanding issues. Results will also be sent to all participating centres who will notify the participants recruited from their site. Participants will be invited to discuss the study results with the local research team.

# 19.  REFERENCE LIST

1. Bouhassira D, et al. Prevalence of chronic pain with neuropathic characteristics in the general population. Pain. 2008; 136:380-387.
2. Finnerup NB, et al. Pharmacotherapy for NeuP in adults: a systematic review and meta-analysis. Lancet Neurol. 2015; 14:162-73.
3. Oyibo SO, Prasad YD, Jackson NJ, Jude EB, Boulton AJ. The relationship between blood glucose excursions and painful diabetic peripheral neuropathy: a pilot study. Diabet Med. 2002;19:870–3.
4. Yang J, Yang X, Zhao D, Wang X, Wei W, Yuan H. Association of time in range, as assessed by continuous glucose monitoring, with painful diabetic polyneuropathy. J Diabetes Investig. 2021;12:828–36.
5. S Tesfaye, ...D Selvarajah. Comparison of amitriptyline supplemented with pregabalin, pregabalin supplemented with amitriptyline, and duloxetine supplemented with pregabalin for the treatment of DPN The Lancet 2022; 400:680-690
6. Dworkin RH et al. Considerations for improving assay sensitivity in chronic pain clinical trials: IMMPACT recommendations. Pain 2012; 153:1148-58
7. Hawker, G.A., et al., Measures of adult pain: Visual Analog Scale for Pain (VAS Pain), Numeric Rating Scale for Pain (NRS Pain), McGill Pain Questionnaire (MPQ), Short-Form McGill Pain Questionnaire (SF-MPQ), Chronic Pain Grade Scale (CPGS), Short Form-36 Bodily Pain Scale (SF-36 BPS), and Measure of Intermittent and Constant Osteoarthritis Pain (ICOAP). Arthritis Care Res (Hoboken), 2011. 63 Suppl 11: p. S240-52.
8. Quilici, S., et al., Meta-analysis of duloxetine vs. pregabalin and gabapentin in the treatment of diabetic peripheral neuropathic pain. BMC Neurol, 2009. 9: p. 6.
9. Agency, E.M. guideline-clinical-development-medicinal-products-intended-treatment-pain-first-version_en.pdf. 2017 10/03/2022]; Available from: <https://www.ema.europa.eu/en/documents/scientific-guideline/guideline-clinical-development-medicinal-products-intended-treatment-pain-first-version_en.pdf>.
10. Richter, R.W., et al., Relief of painful diabetic peripheral neuropathy with pregabalin: a randomized, placebo-controlled trial. J Pain, 2005. 6(4): p. 253-60.
11. Goldstein, D.J., et al., Duloxetine vs. placebo in patients with painful diabetic neuropathy. Pain, 2005. 116(1-2): p. 109-18.
12. Bril V et al. Reliability and validity of the mTCNS in diabetic polyneuropathy. Diabetic Medicine. 2009; 26: 240-6.
13. Zelman DC, Gore M, Dukes E, Tai KS, Brandenburg N. Validation of a modified version of the brief pain inventory for painful diabetic peripheral neuropathy. J Pain Symptom Manage 2005;29:401–410.
14. Feldman, E.L., et al., A practical two-step quantitative clinical and electrophysiological assessment for the diagnosis and staging of diabetic neuropathy. Diabetes Care, 1994. 17(11): p. 1281-9.
15. Group, T.D.R., Factors in development of diabetic neuropathy. Baseline analysis of neuropathy in feasibility phase of Diabetes Control and Complications Trial (DCCT). The DCCT Research Group. Diabetes, 1988. 37(4): p. 476-81.
16. Beck AT, Ward CH, Mendelson M. An inventory for measuring depression. Arch Jen Psych 1961; 4:561-71
17. Ware JE Jr, Sherbourne CD. The MOS 36-item short-form health survey (SF-36). I. Conceptual framework and item selection. Med Care. 1992 Jun;30(6):473-83.
18. Herdman M, Gudex C, Lloyd A, Janssen MF, Kind P, Parkin D, Bonsel G, Badia X (2011). Development and preliminary testing of the new five-level version of EQ-5D (EQ-5D-5L). Quality of Life Research 20(10):1727-36.
19. Joensen LE, Lindgreen P, Olesen K, Nygaard M, Hessler D, Andersen HU, Christensen JO, Kielgast UL, Nørgaard K, Pedersen-Bjergaard U, Willaing I. Validation of the type 1 diabetes distress scale (T1-DDS) in a large Danish cohort: Content validation and psychometric properties. Heliyon. 2023 Mar 18;9(4):e14633.
20. Polonsky WH, Fisher L, Hessler D, Edelman SV. Investigating Hypoglycemic Confidence in Type 1 and Type 2 Diabetes. Diabetes Technol Ther. 2017 Feb;19(2):131-136.
21. Griffin TP, Gallen G, Hartnell S, Crabtree T, Holloway M, Gibb FW, Lumb A, Wilmot EG, Choudhary P, Hussain S. UK's Association of British Clinical Diabetologist's Diabetes Technology Network (ABCD-DTN): Best practice guide for hybrid closed-loop therapy. Diabet Med. 2023 Jul;40(7):e15078.
22. Julious SA. Sample size of 12 per group rule of thumb for a pilot study. Pharm Stat. 2005; 4: 287-91.
