# Supplementary material for: Pain alleviation in diabetic neuropathy using hybrid closed loop insulin pumps (PAINLESS) – A randomised crossover trial protocol
Source: PLoS One. 2026 Sep 8;21(9):e0357573. doi: 10.1371/journal.pone.0357573 (PMC13552766; doi:10.1371/journal.pone.0357573)
Supplement: Supplementary file 6 [file pone.0357573.s007.docx]

Yee Gary Ang

Academic Editor

PLOS One	24/07/2026

Dear Dr Yee Gary Ang,

*re. PONE-D-26-29075*

*Pain Alleviation In diabetic Neuropathy using hybrid cLosEd Loop inSulin pumpS (PAINLESS) – A randomised crossover trial protocol*

We are grateful to the scientific reviewers for reviewing our manuscript and for giving us the opportunity to improve it. We have now addressed the constructive feedback, which has contributed to refining the manuscript. Please find below our responses to each point made by the reviewers.

Yours Sincerely

Dr Simon Berry

**Editor Comments:

We have invited multiple reviewers and 2 reviewers have responded with minor revision.**

We thank you for the positive and constructive peer review, requesting minor revision. We have addressed the comments point-by-point below.

**Journal Requirements:**

**When submitting your revision, we need you to address these additional requirements.**

**1.Please ensure that your manuscript meets PLOS ONE's style requirements, including those for file naming. The PLOS ONE style templates can be found at**

[**https://journals.plos.org/plosone/s/file?id=wjVg/PLOSOne_formatting_sample_main_body.pdf**](https://journals.plos.org/plosone/s/file?id=wjVg/PLOSOne_formatting_sample_main_body.pdf) **and**

[**https://journals.plos.org/plosone/s/file?id=ba62/PLOSOne_formatting_sample_title_authors_affiliations.pdf**](https://track.editorialmanager.com/CL0/https:%2F%2Fjournals.plos.org%2Fplosone%2Fs%2Ffile%3Fid=ba62%2FPLOSOne_formatting_sample_title_authors_affiliations.pdf/1/010f019f73966cbf-3cda7a66-a086-4725-a17c-4c45560d64ae-000000/Guk7ZrzN9MfDJytszedqR6eoISXConpx66wEhgtbyT8=258)

I have updated the headings to sentence case. We believe that the manuscript otherwise meets the formatting requirements but please ask us if there are additional changes that are required.

**2. Thank you for stating the following in the Competing Interests section:**

**" I have read the journal's policy and the authors of this manuscript have the following competing interests: S.B. has received charity-funded travel grants sponsored by Abbott and Eli Lilly. A.I. has received investigator-led funding from Dexcom and Abbott, and has received honoraria from Eli Lilly, Novo Nordisk, Sanofi, Boehringer Ingelheim, Ypsomed, and Diiachi Sankyo as speaker fees. J.E. has received educational / speaker fees from Abbott, Boehringer, Dexcom, Glooko, Insulet, Lilly, Medtronic, Novo Nordisk, Roche, Sanofi, and Ypsomed, and research support from Dexcom. V.K. has received a travel grant from KelCon GmbH, and has received charity-funded travel grants funded by Lilly.  G.S has received honoraria from Procter and Gamble, Viatris, and Eli-Lilly. P.C. has received personal fees from Abbott Diabetes Care, Insulet, Dexcom, Novo Nordisk, AstraZeneca, Medtronic, Roche Diabetes Care, and Sanofi Diabetes, and research funding support from Abbott Diabetes Care, Medtronic, and Novo Nordisk. D.S. has received lecture honoraria from Wörwag Pharma and Grünenthal, unrestricted investigator led grant funding from Withings inc., Abbott Laboratories, Tandem Diabetes and Procter and Gamble, and is an executive committee member of the Novo Nordisk UK Research Foundation. A.P., A.G., R.G., S.M.G. and M.H. have no relevant disclosures to declare.”**

**We note that one or more of the authors are employed by a commercial company: “Abbott, Eli Lilly, Dexcom, Novo Nordisk, Sanofi, Boehringer Ingelheim, Ypsomed, Diiachi Sankyo, KelCon GmbH, Procter and Gamble, Viatris, Abbott Diabetes Care, Insulet, AstraZeneca, Medtronic, Roche Diabetes Care, and Sanofi Diabetes, Wörwag Pharma and Grünenthal, Withings inc., Abbott Laboratories, Tandem Diabetes Novo Nordisk UK Research Foundation”**

**1. Please provide an amended Funding Statement declaring this commercial affiliation, as well as a statement regarding the Role of Funders in your study. If the funding organization did not play a role in the study design, data collection and analysis, decision to publish, or preparation of the manuscript and only provided financial support in the form of authors' salaries and/or research materials, please review your statements relating to the author contributions, and ensure you have specifically and accurately indicated the role(s) that these authors had in your study. You can update author roles in the Author Contributions section of the online submission form. Please also include the following statement within your amended Funding Statement.**

**“The funder provided support in the form of salaries for authors “S.B., J.E., A.I., V.K., G.S., P.C., and D.S.,” but did not have any additional role in the study design, data collection and analysis, decision to publish, or preparation of the manuscript. The specific roles of these authors are articulated in the ‘author contributions’ section.”**

**If your commercial affiliation did play a role in your study, please state and explain this role within your updated Funding Statement.**

Thank you for your comment. Unfortunately, we believe there has been a misunderstanding. No authors have received salaries from any of these companies. The details of their individual competing interests are detailed in the statement but we can confirm that none of the authors are employed by a commercial company. Most of these competing interests are either honoraria for speaking or research support paid to their host universities and are unrelated to this study. The only funder of this study is Tandem Diabetes Care. As detailed in the funding statement, we can confirm that “The Funder of the trial and other suppliers have had no role in the trial design, data collection, data analysis or data interpretation.” I have added a statement at the end of the competing interests “None of these competing interests have had a role in the trial design, data collection, data analysis or data interpretation of this study” to re-affirm this statement. Please note the updated funding statement and competing interests is at the end of this cover letter.

**2. Please also provide an updated Competing Interests Statement declaring this commercial affiliation along with any other relevant declarations relating to employment, consultancy, patents, products in development, or marketed products, etc.**

**Within your Competing Interests Statement, please confirm that this commercial affiliation does not alter your adherence to all PLOS ONE policies on sharing data and materials by including the following statement: "This does not alter our adherence to  PLOS ONE policies on sharing data and materials.” (as detailed online in our guide for authors** [**http://journals.plos.org/plosone/s/competing-interests**](http://journals.plos.org/plosone/s/competing-interests)**) . If this adherence statement is not accurate and  there are restrictions on sharing of data and/or materials, please state these. Please note that we cannot proceed with consideration of your article until this information has been declared.**

**Please include both an updated Funding Statement and Competing Interests Statement in your cover letter. We will change the online submission form on your behalf.**

Many thanks for your comment. I have added this statement to the competing interests section. Please note the updated funding statement and competing interests is at the end of this cover letter.

**3. Please provide a complete Data Availability Statement in the submission form, ensuring you include all necessary access information or a reason for why you are unable to make your data freely accessible. If your research concerns only data provided within your submission, please write "All data are in the manuscript and/or supporting information files" as your Data Availability Statement.**

I have updated the data availability statement in the submission form. As this is a protocol paper, with recruitment ongoing, and data analysis not started, no data is available at present.

**4. When completing the data availability statement of the submission form, you indicated that you will make your data available on acceptance. We strongly recommend all authors decide on a data sharing plan before acceptance, as the process can be lengthy and hold up publication timelines. Please note that, though access restrictions are acceptable now, your entire data will need to be made freely accessible if your manuscript is accepted for publication. This policy applies to all data except where public deposition would breach compliance with the protocol approved by your research ethics board. If you are unable to adhere to our open data policy, please kindly revise your statement to explain your reasoning and we will seek the editor's input on an exemption. Please be assured that, once you have provided your new statement, the assessment of your exemption will not hold up the peer review process.**

As this is a protocol paper, we have included all data available at present. I have updated this in the data availability statement.

**5. We note that the original protocol that you have uploaded as a Supporting Information file contains an institutional logo. As this logo is likely copyrighted, we ask that you please remove it from this file and upload an updated version upon resubmission.**

Thank you for raising this. I have removed the institutional logo from the relevant supporting files.

**6. If the reviewer comments include a recommendation to cite specific previously published works, please review and evaluate these publications to determine whether they are relevant and should be cited. There is no requirement to cite these works unless the editor has indicated otherwise.**

The reviewers have not asked for specific citations. Two citations have been added to address reviewers’ comments but neither was directly requested.

**7. Please review your reference list to ensure that it is complete and correct. If you have cited papers that have been retracted, please include the rationale for doing so in the manuscript text, or remove these references and replace them with relevant current references. Any changes to the reference list should be mentioned in the rebuttal letter that accompanies your revised manuscript. If you need to cite a retracted article, indicate the article’s retracted status in the References list and also include a citation and full reference for the retraction notice.**

Thank you for your comment. We have added one reference to explain the minimisation method and a further to explain why only people with type 1 diabetes were included. No other references have been altered or removed due to retraction.

**Reviewer #1:**

**This is a protocol of a crossover study. It is well-written and I have only some minor statistical comments:
1. Analysis will be conducted by a statistician blinded to treatment allocation. I'm not sure how a statistician can do an analysis of a treatment effect without including the treatment allocation in the analysis!**

Many thanks for your constructive review. I will now address each comment point-by-point. The statistician will be blinded to the treatment allocation as each intervention will be given a code e.g. treatment A or treatment B. We have added this to the text lines 353 “Analyses will be conducted by a statistician blinded to treatment sequence (the treatment sequence will be coded)”.

**2. Minimization is a broad term that could include Taves's non-randomized procedure or Pocock-Simon's randomized procedure. You do not specify or provide a reference. See Rosenberger and Lachin (2016, Randomization in Clinical Trials, Chapter 9).**

Thank you for pointing this out. The randomization procedure uses Pocock-Simon’s randomized procedure. On lines 259-260, the sentence now reads “The randomisation will use the Pocock-Simon minimisation algorithm to ensure balance in the treatment allocation taking into account centre, age (18-39, 40-59, 60+) and gender at birth [28]”.

**3. The general inference philosophy following minimization-type procedures is that the minimized variables should be included in the model to achieve valid inference (this is mentioned in the same chapter in the reference above). You do not seem to be doing this.**

Thank you for this comment. We agree that variables used in the minimisation procedure should be included in the analysis model. We have clarified in the Methods that the primary analysis will be adjusted for centre, age group and gender at birth on lines 366-367 “The model will include treatment arm, period, sequence, gender at birth, centre and age group as fixed effects and participant as a random effect to account for the repeated measures design.”

**4. Random effects models are used with no description of the covariance structure, what diagnostics will be used to verify model assumptions, or what the underlying distribution of the outcome variable (NRS) is.**

Thank you for this comment. We have expanded the statistical analysis section to describe the mixed-effects model in greater detail, including the covariance structure, the planned model diagnostic assessment and the rationale for treating the NRS score as a continuous outcome (lines 368-371). “Model assumptions will be assessed using residual versus fitted plots and Q-Q plots of residuals. The NRS pain score (0-10) will be analysed as a continuous outcome, consistent with established practice in chronic pain trials. If model assumptions are violated, sensitivity analyses using alternative modelling approaches will be considered.”

**5. 49 seems an "odd" number for a 2 arm 1:1 clinical trial. I guess you can split one of the patients in half.**

Thank you for this comment. We have updated the description to explain that 49 is an approximate number for enrolment to achieve 40 randomised participants, accounting for dropout during the screening period (lines 127-128) “To account for anticipated dropout and screen failure rate, the PAINLESS study will enrol approximately 49 participants in the screening period to achieve a randomised sample size of 40 participants in total across two research sites in the United Kingdom – Sheffield Teaching Hospitals National Health Service (NHS) Foundation Trust and University Hospitals of Leicester NHS Trust.”

**Reviewer #2**

**In the protocol, use of a hybrid closed loop (HCL) system is compared with "standard care", which is the diabetes management used by the participant on entering the study. The eligibility and exclusion criteria do not exclude somebody from the study if they are already using a HCL system. Given the protocol is designed using the assumption that glycaemic variability will be less using an HCL system v standard care, why is this not excluded?**

Thank you for this comment and for pointing this out. To this date, no people already using HCL have enrolled in the study. However, we will incorporate a change in the exclusion criteria into an upcoming ethics amendment to exclude those already using HCL systems, only including those on multiple daily injections or open loop insulin pump systems.  **Glycaemic variability will be compared between the groups using the measurement of percentage coefficient of variation (%CV), a standard continuous glucose monitoring (CGM) reporting measure. Why is the comparison of this measure between standard care and HCL use not more prominent in the analysis plan? It would seem that this should be carried out prior to other analyses, as if there is no difference in glycaemic variability between the conditions then the stated hypothesis cannot be tested. There may be other reasons that HCL treatment improves symptoms, and therefore the experiment remains valid even if this specific hypothesis is not tested directly, but that should be made clear.**

Thank you for the comment. We agree that it is important to establish whether a change in glucose variability has been achieved. We will continue to analyse the results regardless of whether a change in glycaemic variability has been identified, in order to determine whether HCL treatment improves pain. To address this point in the manuscript, we have added the following to the description of the statistical analysis plan lines 360-364 “As a preliminary step prior to the primary analysis, the change in the coefficient of variation (CV) between the control intervention period and HCL intervention period will be evaluated to assess intervention effect on glycaemic variability. The primary analysis will proceed regardless of whether a statistically significant difference in glucose CV is observed.”
 **People with type 2 diabetes are excluded from this study. This should be justified directly, as there is evidence that HCL can improve glycaemia in type 2 diabetes as well. I suspect the reason is that HCL treatment is currently funded in England under NICE TA 943 for eligible adults with type 1 diabetes and therefore there is the potential for participants to continue with the treatment after completion of the study, which would not currently be the case for type 2 diabetes. However, this should be made explicit.**

Thank you for the comment. The decision to focus on individuals with type 1 diabetes had two main reasons. One was a pragmatic decision based on licensing. At the time of planning this study, the tandem t:slim X2 was not licensed for people with type 2 diabetes in the United Kingdom (it was only approved in mid-2026). Secondly, the pathophysiology of painful peripheral neuropathy differs between those with type 1 and type 2 diabetes, with metabolic syndrome playing a more prominent role in type 2 diabetes and glucose excursions a more prominent role in type 1 diabetes. Therefore, people with type 1 diabetes hypothetically may benefit more when reducing glycaemic variability. As a result of the differing pathophysiology, we have chosen to keep a homogenous type 1 diabetes population rather than incorporating both type 1 and 2 in one study. We definitely agree that future research should also address this question in people with type 2 diabetes. For the readers, we have explicitly stated on lines 114-116 that people with type 2 diabetes are excluded “People with type 2 diabetes are not included as the pathophysiology of PDN differs, with greater influence of metabolic syndrome and dyslipidaemia [20].”  **Note that the image of a CGM device included in the PIS is of a Dexcom G6 sensor - this should be replaced with an image of the correct sensor.**

Thank you for pointing this out. The image has been updated and we will update the PIS in an ethics amendment. **Minor comment - introduction paragraph 2 line 68, the abbreviation pwT1D is used without explanation - this should be explained before abbreviation.**

Thank you for raising this. The abbreviation has now been explained.

**Funding**

The work is supported by a grant from Tandem Diabetes Care, Inc (San Diego, USA). Tandem Diabetes Care, Inc (San Diego, USA) also provided t-slim X2 insulin pumps with Control-IQ technology and consumables for study participants. Dexcom, Inc (San Diego, USA) provided Dexcom G7 sensors for study participants. Withings (Issy-les-Moulineaux, France) provided activity and sleep trackers for study participants. VibroSense Dynamics AB (Malmo, Sweden) provided one VibroSense Meter II per site. The Funder of the trial and other suppliers have had no role in the trial design, data collection, data analysis or data interpretation.

**Competing interests**

S.B. has received charity-funded travel grants sponsored by Abbott and Eli Lilly. A.I. has received investigator-led funding from Dexcom and Abbott, and has received honoraria from Eli Lilly, Novo Nordisk, Sanofi, Boehringer Ingelheim, Ypsomed, and Diiachi Sankyo as speaker fees. J.E. has received educational / speaker fees from Abbott, Boehringer, Dexcom, Glooko, Insulet, Lilly, Medtronic, Novo Nordisk, Roche, Sanofi, and Ypsomed, and research support from Dexcom. V.K. has received a travel grant from KelCon GmbH, and has received charity-funded travel grants funded by Lilly. G.S has received honoraria from Procter and Gamble, Viatris, and Eli-Lilly. P.C. has received personal fees from Abbott Diabetes Care, Insulet, Dexcom, Novo Nordisk, AstraZeneca, Medtronic, Roche Diabetes Care, and Sanofi Diabetes, and research funding support from Abbott Diabetes Care, Medtronic, and Novo Nordisk. D.S. has received lecture honoraria from Wörwag Pharma and Grünenthal, unrestricted investigator led grant funding from Withings inc., Abbott Laboratories, Tandem Diabetes and Procter and Gamble, and is an executive committee member of the Novo Nordisk UK Research Foundation. A.P., A.G., R.G., S.M.G. and M.H. have no relevant disclosures to declare. None of these competing interests have had a role in the trial design, data collection, data analysis or data interpretation of this study. These competing interests do not alter our adherence to PLOS ONE policies on sharing data and materials.
