# Supplementary material for: Pain alleviation in diabetic neuropathy using hybrid closed loop insulin pumps (PAINLESS) – A randomised crossover trial protocol
Source: PLoS One. 2026 Sep 8;21(9):e0357573. doi: 10.1371/journal.pone.0357573 (PMC13552766; doi:10.1371/journal.pone.0357573)
Supplement: Supplementary file 7 [file pone.0357573.s002.doc]

**Pain Diary Day 1**

Introduction:

- Please complete this record each morning within one hour of rising.
- Please tick the box at the point which describes your average pain over the past 24 hours

A) **Average Pain**

### 0

### 1

### 2

### 3

### 4

### 5

### 6

### 7

### 8

### 9

### 10

**Pain as bad as it could be**

### No Pain

**B) Which time in the day was pain most severe:**

**Night**

**2300-0700**

**Afternoon**

**1300-1700**

### Morning

**0800-1200**

**Evening**

**1800-2200**

C) **Please record any rescue pain medication you have used in the last 24 hours:**

**Sleep Diary Day 1**

Introduction:

- Please complete this record each morning within one hour of rising.
- Please tick the box which best describes your sleep last night.


##### D)  Sleep Disturbance

Please indicate below the number of times you woke last night due to your symptoms:

Please tick one box only.

**None	Once	Twice	More than twice**

##### E) Sleep Quality

**Very Bad	Bad	Satisfactory	Good	Very Good**
