# Supplementary material for: Pain alleviation in diabetic neuropathy using hybrid closed loop insulin pumps (PAINLESS) – A randomised crossover trial protocol
Source: PLoS One. 2026 Sep 8;21(9):e0357573. doi: 10.1371/journal.pone.0357573 (PMC13552766; doi:10.1371/journal.pone.0357573)
Supplement: Peer review history

# Peer review: Decision Letter 0

**DOI:** 10.1371/journal.pone.0357573.r001

**Authors:** Yee Gary Ang

18 Jul 2026

Dear Dr. Berry,

Thank you for submitting your manuscript to PLOS ONE. After careful consideration, we feel that it has merit but does not fully meet PLOS ONE’s publication criteria as it currently stands. Therefore, we invite you to submit a revised version of the manuscript that addresses the points raised during the review process.

Please submit your revised manuscript by Aug 02 2026 11:59PM. If you will need more time than this to complete your revisions, please reply to this message or contact the journal office at plosone@plos.org. When you're ready to submit your revision, log on to https://www.editorialmanager.com/pone/ and select the 'Submissions Needing Revision' folder to locate your manuscript file.

- A letter that responds to each point raised by the academic editor and reviewer(s). You should upload this letter as a separate file labeled 'Response to Reviewers'.
- A marked-up copy of your manuscript that highlights changes made to the original version. You should upload this as a separate file labeled 'Revised Manuscript with Track Changes'.
- An unmarked version of your revised paper without tracked changes. You should upload this as a separate file labeled 'Manuscript'.

If you would like to make changes to your financial disclosure, please include your updated statement in your cover letter. Guidelines for resubmitting your figure files are available below the reviewer comments at the end of this letter.

If applicable, we recommend that you deposit your laboratory protocols in protocols.io to enhance the reproducibility of your results. Protocols.io assigns your protocol its own identifier (DOI) so that it can be cited independently in the future. For instructions see: https://journals.plos.org/plosone/s/submission-guidelines#loc-laboratory-protocols. Additionally, PLOS ONE offers an option for publishing peer-reviewed Lab Protocol articles, which describe protocols hosted on protocols.io. Read more information on sharing protocols at https://plos.org/protocols?utm_medium=editorial-email&utm_source=authorletters&utm_campaign=protocols.

As the corresponding author, your ORCID iD is verified in the submission system and will appear in the published article. PLOS supports the use of ORCID, and we encourage all coauthors to register for an ORCID iD and use it as well. Please encourage your coauthors to verify their ORCID iD within the submission system before final acceptance, as unverified ORCID iDs will not appear in the published article. *Only*  the individual author can complete the verification step; PLOS staff *cannot*  verify ORCID iDs on behalf of authors.

We look forward to receiving your revised manuscript.

Kind regards,

Yee Gary Ang, MBBS MPH

Academic Editor

PLOS One

Journal Requirements:

When submitting your revision, we need you to address these additional requirements.

1.Please ensure that your manuscript meets PLOS ONE's style requirements, including those for file naming. The PLOS ONE style templates can be found at

https://journals.plos.org/plosone/s/file?id=wjVg/PLOSOne_formatting_sample_main_body.pdf and

https://journals.plos.org/plosone/s/file?id=ba62/PLOSOne_formatting_sample_title_authors_affiliations.pdf

2. Thank you for stating the following in the Competing Interests section:

" I have read the journal's policy and the authors of this manuscript have the following competing interests: S.B. has received charity-funded travel grants sponsored by Abbott and Eli Lilly. A.I. has received investigator-led funding from Dexcom and Abbott, and has received honoraria from Eli Lilly, Novo Nordisk, Sanofi, Boehringer Ingelheim, Ypsomed, and Diiachi Sankyo as speaker fees. J.E. has received educational / speaker fees from Abbott, Boehringer, Dexcom, Glooko, Insulet, Lilly, Medtronic, Novo Nordisk, Roche, Sanofi, and Ypsomed, and research support from Dexcom. V.K. has received a travel grant from KelCon GmbH, and has received charity-funded travel grants funded by Lilly.  G.S has received honoraria from Procter and Gamble, Viatris, and Eli-Lilly. P.C. has received personal fees from Abbott Diabetes Care, Insulet, Dexcom, Novo Nordisk, AstraZeneca, Medtronic, Roche Diabetes Care, and Sanofi Diabetes, and research funding support from Abbott Diabetes Care, Medtronic, and Novo Nordisk. D.S. has received lecture honoraria from Wörwag Pharma and Grünenthal, unrestricted investigator led grant funding from Withings inc., Abbott Laboratories, Tandem Diabetes and Procter and Gamble, and is an executive committee member of the Novo Nordisk UK Research Foundation. A.P., A.G., R.G., S.M.G. and M.H. have no relevant disclosures to declare.”

We note that one or more of the authors are employed by a commercial company: “Abbott, Eli Lilly, Dexcom, Novo Nordisk, Sanofi, Boehringer Ingelheim, Ypsomed, Diiachi Sankyo, KelCon GmbH, Procter and Gamble, Viatris, Abbott Diabetes Care, Insulet, AstraZeneca, Medtronic, Roche Diabetes Care, and Sanofi Diabetes, Wörwag Pharma and Grünenthal, Withings inc., Abbott Laboratories, Tandem Diabetes Novo Nordisk UK Research Foundation”

1. Please provide an amended Funding Statement declaring this commercial affiliation, as well as a statement regarding the Role of Funders in your study. If the funding organization did not play a role in the study design, data collection and analysis, decision to publish, or preparation of the manuscript and only provided financial support in the form of authors' salaries and/or research materials, please review your statements relating to the author contributions, and ensure you have specifically and accurately indicated the role(s) that these authors had in your study. You can update author roles in the Author Contributions section of the online submission form.

Please also include the following statement within your amended Funding Statement.

“The funder provided support in the form of salaries for authors “S.B., J.E., A.I., V.K., G.S., P.C., and D.S.,” but did not have any additional role in the study design, data collection and analysis, decision to publish, or preparation of the manuscript. The specific roles of these authors are articulated in the ‘author contributions’ section.”

If your commercial affiliation did play a role in your study, please state and explain this role within your updated Funding Statement.

2. Please also provide an updated Competing Interests Statement declaring this commercial affiliation along with any other relevant declarations relating to employment, consultancy, patents, products in development, or marketed products, etc.

Within your Competing Interests Statement, please confirm that this commercial affiliation does not alter your adherence to all PLOS ONE policies on sharing data and materials by including the following statement: "This does not alter our adherence to  PLOS ONE policies on sharing data and materials.” (as detailed online in our guide for authors http://journals.plos.org/plosone/s/competing-interests) . If this adherence statement is not accurate and  there are restrictions on sharing of data and/or materials, please state these. Please note that we cannot proceed with consideration of your article until this information has been declared.

Please include both an updated Funding Statement and Competing Interests Statement in your cover letter. We will change the online submission form on your behalf.

3. Please provide a complete Data Availability Statement in the submission form, ensuring you include all necessary access information or a reason for why you are unable to make your data freely accessible. If your research concerns only data provided within your submission, please write "All data are in the manuscript and/or supporting information files" as your Data Availability Statement.

4. When completing the data availability statement of the submission form, you indicated that you will make your data available on acceptance. We strongly recommend all authors decide on a data sharing plan before acceptance, as the process can be lengthy and hold up publication timelines. Please note that, though access restrictions are acceptable now, your entire data will need to be made freely accessible if your manuscript is accepted for publication. This policy applies to all data except where public deposition would breach compliance with the protocol approved by your research ethics board. If you are unable to adhere to our open data policy, please kindly revise your statement to explain your reasoning and we will seek the editor's input on an exemption. Please be assured that, once you have provided your new statement, the assessment of your exemption will not hold up the peer review process.

5. We note that the original protocol that you have uploaded as a Supporting Information file contains an institutional logo. As this logo is likely copyrighted, we ask that you please remove it from this file and upload an updated version upon resubmission.

6. If the reviewer comments include a recommendation to cite specific previously published works, please review and evaluate these publications to determine whether they are relevant and should be cited. There is no requirement to cite these works unless the editor has indicated otherwise.

7. Please review your reference list to ensure that it is complete and correct. If you have cited papers that have been retracted, please include the rationale for doing so in the manuscript text, or remove these references and replace them with relevant current references. Any changes to the reference list should be mentioned in the rebuttal letter that accompanies your revised manuscript. If you need to cite a retracted article, indicate the article’s retracted status in the References list and also include a citation and full reference for the retraction notice.

Additional Editor Comments:

**We have invited multiple reviewers and 2 reviewers have responded with minor revision.**

**Please see and resubmit if you are able to address the comments**

[Note: HTML markup is below. Please do not edit.]

Reviewer's Responses to Questions

**Comments to the Author**

1. Does the manuscript provide a valid rationale for the proposed study, with clearly identified and justified research questions?

Reviewer #1: Yes

Reviewer #2: Yes

**********

2. Is the protocol technically sound and planned in a manner that will lead to a meaningful outcome and allow testing the stated hypotheses?

Reviewer #1: Yes

Reviewer #2: Yes

**********

3. Is the methodology feasible and described in sufficient detail to allow the work to be replicable?

Reviewer #1: Yes

Reviewer #2: Yes

**********

4. Have the authors described where all data underlying the findings will be made available when the study is complete?

The PLOS Data policy

Reviewer #1: Yes

Reviewer #2: Yes

**********

5. Is the manuscript presented in an intelligible fashion and written in standard English?

Reviewer #1: Yes

Reviewer #2: Yes

**********

Please use the space provided to explain your answers to the questions above and, if applicable, provide comments about issues authors must address before this protocol can be accepted for publication. You may also include additional comments for the author, including concerns about research or publication ethics.

You may also provide optional suggestions and comments to authors that they might find helpful in planning their study.

Reviewer #1: This is a protocol of a crossover study. It is well-written and I have only some minor statistical comments:

1. Analysis will be conducted by a statistician blinded to treatment allocation. I'm not sure how a statistician can do an analysis of a treatment effect without including the treatment allocation in the analysis!

2. Minimization is a broad term that could include Taves's non-randomized procedure or Pocock-Simon's randomized procedure. You do not specify or provide a reference. See Rosenberger and Lachin (2016, Randomization in Clinical Trials, Chapter 9).

3. The general inference philosophy following minimization-type procedures is that the minimized variables should be included in the model to achieve valid inference (this is mentioned in the same chapter in the reference above). You do not seem to be doing this.

4. Random effects models are used with no description of the covariance structure, what diagnostics will be used to verify model assumptions, or what the underlying distribution of the outcome variable (NRS) is.

5. 49 seems an "odd" number for a 2 arm 1:1 clinical trial. I guess you can split one of the patients in half.

Reviewer #2: In the protocol, use of a hybrid closed loop (HCL) system is compared with "standard care", which is the diabetes management used by the participant on entering the study. The eligibility and exclusion criteria do not exclude somebody from the study if they are already using a HCL system. Given the protocol is designed using the assumption that glycaemic variability will be less using an HCL system v standard care, why is this not excluded?

Glycaemic variability will be compared between the groups using the measurement of percentage coefficient of variation (%CV), a standard continuous glucose monitoring (CGM) reporting measure. Why is the comparison of this measure between standard care and HCL use not more prominent in the analysis plan? It would seem that this should be carried out prior to other analyses, as if there is no difference in glycaemic variability between the conditions then the stated hypothesis cannot be tested. There may be other reasons that HCL treatment improves symptoms, and therefore the experiment remains valid even if this specific hypothesis is not tested directly, but that should be made clear.

People with type 2 diabetes are excluded from this study. This should be justified directly, as there is evidence that HCL can improve gllycaemia in type 2 diabetes as well. I suspect the reason is that HCL treatment is currently funded in England under NICE TA 943 for eligible adults with type 1 diabetes and therefore there is the potential for participants to continue with the treatment after completion of the study, which would not currently be the case for type 2 diabetes. However, this should be made explicit.

Note that the image of a CGM device included in the PIS is of a Dexcom G6 sensor - this should be replaced with an image of the correct sensor.

Minro comment - introduction paragraph 2 line 68, the abbreviation pwT1D is used without explanation - this should be explained before abbreviation.

**********

what does this mean?). If published, this will include your full peer review and any attached files.

If you choose “no”, your identity will remain anonymous but your review may still be made public.

**Do you want your identity to be public for this peer review?**  For information about this choice, including consent withdrawal, please see our Privacy Policy

Reviewer #1: No

Reviewer #2: No

**********

[NOTE: If reviewer comments were submitted as an attachment file, they will be attached to this email and accessible via the submission site. Please log into your account, locate the manuscript record, and check for the action link "View Attachments". If this link does not appear, there are no attachment files.]

To ensure your figures meet our technical requirements, please review our figure guidelines: https://journals.plos.org/plosone/s/figures

You may also use PLOS’s free figure tool, NAAS, to help you prepare publication quality figures: https://journals.plos.org/plosone/s/figures#loc-tools-for-figure-preparation.

NAAS will assess whether your figures meet our technical requirements by comparing each figure against our figure specifications.

# Author comment: Author response to Decision Letter 1

**DOI:** 10.1371/journal.pone.0357573.r002

24 Jul 2026

Reviewer #1:

This is a protocol of a crossover study. It is well-written and I have only some minor statistical comments:

1. Analysis will be conducted by a statistician blinded to treatment allocation. I'm not sure how a statistician can do an analysis of a treatment effect without including the treatment allocation in the analysis!

Many thanks for your constructive review. I will now address each comment point-by-point. The statistician will be blinded to the treatment allocation as each intervention will be given a code e.g. treatment A or treatment B. We have added this to the text lines 353 “Analyses will be conducted by a statistician blinded to treatment sequence (the treatment sequence will be coded)”.

2. Minimization is a broad term that could include Taves's non-randomized procedure or Pocock-Simon's randomized procedure. You do not specify or provide a reference. See Rosenberger and Lachin (2016, Randomization in Clinical Trials, Chapter 9).

Thank you for pointing this out. The randomization procedure uses Pocock-Simon’s randomized procedure. On lines 259-260, the sentence now reads “The randomisation will use the Pocock-Simon minimisation algorithm to ensure balance in the treatment allocation taking into account centre, age (18-39, 40-59, 60+) and gender at birth [28]”.

3. The general inference philosophy following minimization-type procedures is that the minimized variables should be included in the model to achieve valid inference (this is mentioned in the same chapter in the reference above). You do not seem to be doing this.

Thank you for this comment. We agree that variables used in the minimisation procedure should be included in the analysis model. We have clarified in the Methods that the primary analysis will be adjusted for centre, age group and gender at birth on lines 366-367 “The model will include treatment arm, period, sequence, gender at birth, centre and age group as fixed effects and participant as a random effect to account for the repeated measures design.”

4. Random effects models are used with no description of the covariance structure, what diagnostics will be used to verify model assumptions, or what the underlying distribution of the outcome variable (NRS) is.

Thank you for this comment. We have expanded the statistical analysis section to describe the mixed-effects model in greater detail, including the covariance structure, the planned model diagnostic assessment and the rationale for treating the NRS score as a continuous outcome (lines 368-371). “Model assumptions will be assessed using residual versus fitted plots and Q-Q plots of residuals. The NRS pain score (0-10) will be analysed as a continuous outcome, consistent with established practice in chronic pain trials. If model assumptions are violated, sensitivity analyses using alternative modelling approaches will be considered.”

5. 49 seems an "odd" number for a 2 arm 1:1 clinical trial. I guess you can split one of the patients in half.

Thank you for this comment. We have updated the description to explain that 49 is an approximate number for enrolment to achieve 40 randomised participants, accounting for dropout during the screening period (lines 127-128) “To account for anticipated dropout and screen failure rate, the PAINLESS study will enrol approximately 49 participants in the screening period to achieve a randomised sample size of 40 participants in total across two research sites in the United Kingdom – Sheffield Teaching Hospitals National Health Service (NHS) Foundation Trust and University Hospitals of Leicester NHS Trust.”

Reviewer #2

In the protocol, use of a hybrid closed loop (HCL) system is compared with "standard care", which is the diabetes management used by the participant on entering the study. The eligibility and exclusion criteria do not exclude somebody from the study if they are already using a HCL system. Given the protocol is designed using the assumption that glycaemic variability will be less using an HCL system v standard care, why is this not excluded?

Thank you for this comment and for pointing this out. To this date, no people already using HCL have enrolled in the study. However, we will incorporate a change in the exclusion criteria into an upcoming ethics amendment to exclude those already using HCL systems, only including those on multiple daily injections or open loop insulin pump systems.

Glycaemic variability will be compared between the groups using the measurement of percentage coefficient of variation (%CV), a standard continuous glucose monitoring (CGM) reporting measure. Why is the comparison of this measure between standard care and HCL use not more prominent in the analysis plan? It would seem that this should be carried out prior to other analyses, as if there is no difference in glycaemic variability between the conditions then the stated hypothesis cannot be tested. There may be other reasons that HCL treatment improves symptoms, and therefore the experiment remains valid even if this specific hypothesis is not tested directly, but that should be made clear.

Thank you for the comment. We agree that it is important to establish whether a change in glucose variability has been achieved. We will continue to analyse the results regardless of whether a change in glycaemic variability has been identified, in order to determine whether HCL treatment improves pain. To address this point in the manuscript, we have added the following to the description of the statistical analysis plan lines 360-364 “As a preliminary step prior to the primary analysis, the change in the coefficient of variation (CV) between the control intervention period and HCL intervention period will be evaluated to assess intervention effect on glycaemic variability. The primary analysis will proceed regardless of whether a statistically significant difference in glucose CV is observed.”

People with type 2 diabetes are excluded from this study. This should be justified directly, as there is evidence that HCL can improve glycaemia in type 2 diabetes as well. I suspect the reason is that HCL treatment is currently funded in England under NICE TA 943 for eligible adults with type 1 diabetes and therefore there is the potential for participants to continue with the treatment after completion of the study, which would not currently be the case for type 2 diabetes. However, this should be made explicit.

Thank you for the comment. The decision to focus on individuals with type 1 diabetes had two main reasons. One was a pragmatic decision based on licensing. At the time of planning this study, the tandem t:slim X2 was not licensed for people with type 2 diabetes in the United Kingdom (it was only approved in mid-2026). Secondly, the pathophysiology of painful peripheral neuropathy differs between those with type 1 and type 2 diabetes, with metabolic syndrome playing a more prominent role in type 2 diabetes and glucose excursions a more prominent role in type 1 diabetes. Therefore, people with type 1 diabetes hypothetically may benefit more when reducing glycaemic variability. As a result of the differing pathophysiology, we have chosen to keep a homogenous type 1 diabetes population rather than incorporating both type 1 and 2 in one study. We definitely agree that future research should also address this question in people with type 2 diabetes. For the readers, we have explicitly stated on lines 114-116 that people with type 2 diabetes are excluded “People with type 2 diabetes are not included as the pathophysiology of PDN differs, with greater influence of metabolic syndrome and dyslipidaemia [20].”

Note that the image of a CGM device included in the PIS is of a Dexcom G6 sensor - this should be replaced with an image of the correct sensor.

Thank you for pointing this out. The image has been updated and we will update the PIS in an ethics amendment.

Minor comment - introduction paragraph 2 line 68, the abbreviation pwT1D is used without explanation - this should be explained before abbreviation.

Thank you for raising this. The abbreviation has now been explained.

Attachment Submitted filename: Response to reviewers.docx

# Peer review: Decision Letter 1

**DOI:** 10.1371/journal.pone.0357573.r003

**Authors:** Yee Gary Ang

19 Aug 2026

Pain Alleviation In diabetic Neuropathy using hybrid cLosEd Loop inSulin pumpS (PAINLESS) – A randomised crossover trial protocol

PONE-D-26-29075R1

Dear Dr. Berry,

We’re pleased to inform you that your manuscript has been judged scientifically suitable for publication and will be formally accepted for publication once it meets all outstanding technical requirements.

Within one week, you’ll receive an e-mail detailing the required amendments. When these have been addressed, you’ll receive a formal acceptance letter and your manuscript will be scheduled for publication.

An invoice will be generated when your article is formally accepted. Please note, if your institution has a publishing partnership with PLOS and your article meets the relevant criteria, all or part of your publication costs will be covered. Please make sure your user information is up-to-date by logging into Editorial Manager at Editorial Manager® and clicking the ‘Update My Information' link at the top of the page. For questions related to billing, please contact billing support.

If your institution or institutions have a press office, please notify them about your upcoming paper to help maximize its impact. If they’ll be preparing press materials, please inform our press team as soon as possible -- no later than 48 hours after receiving the formal acceptance. Your manuscript will remain under strict press embargo until 2 pm Eastern Time on the date of publication. For more information, please contact onepress@plos.org.

Kind regards,

Yee Gary Ang, MBBS MPH

Academic Editor

PLOS One

Additional Editor Comments (optional):

Reviewers' comments:

Reviewer's Responses to Questions

**Comments to the Author**

1. Does the manuscript provide a valid rationale for the proposed study, with clearly identified and justified research questions?

Reviewer #1: Yes

Reviewer #2: Yes

**********

2. Is the protocol technically sound and planned in a manner that will lead to a meaningful outcome and allow testing the stated hypotheses?

Reviewer #1: Yes

Reviewer #2: Yes

**********

3. Is the methodology feasible and described in sufficient detail to allow the work to be replicable?

Reviewer #1: Yes

Reviewer #2: Yes

**********

4. Have the authors described where all data underlying the findings will be made available when the study is complete?

The PLOS Data policy

Reviewer #1: Yes

Reviewer #2: Yes

**********

5. Is the manuscript presented in an intelligible fashion and written in standard English?

Reviewer #1: Yes

Reviewer #2: Yes

**********

Please use the space provided to explain your answers to the questions above and, if applicable, provide comments about issues authors must address before this protocol can be accepted for publication. You may also include additional comments for the author, including concerns about research or publication ethics.

You may also provide optional suggestions and comments to authors that they might find helpful in planning their study.

Reviewer #1: All comments have been addressed, although I still don't understand why the study statistician needs to be blinded to treatment assignment. Won't he/she write the results section of your papers? How do you do that if you don't know which treatment is which? Overkill. Statisticians do not participate in patient recruitment and are bound by a code of ethics to not divulge anything during the trial.

Reviewer #2: Many thanks for the opportunity to review the revised manuscript. My previous comments have been fully addressed.

I just have a coupe of very minor comments on the revised draft.

P5 line 75 – people with diabetes prefer us to avoid the term “glycaemic control” – please consider an alternative such a “glucose levels” or “glycaemia”

P8 line 144 – Dexcom G7 is indicated for use on the abdomen as well as the upper arm in the UK

**********

what does this mean?). If published, this will include your full peer review and any attached files.

If you choose “no”, your identity will remain anonymous but your review may still be made public.

**Do you want your identity to be public for this peer review?**  For information about this choice, including consent withdrawal, please see our Privacy Policy

Reviewer #1: No

Reviewer #2: No

**********

# Editor report: Acceptance letter

**DOI:** 10.1371/journal.pone.0357573.r004

**Authors:** Yee Gary Ang

PONE-D-26-29075R1

PLOS One

Dear Dr. Berry,

I'm pleased to inform you that your manuscript has been deemed suitable for publication in PLOS One. Congratulations! Your manuscript is now being handed over to our production team.

At this stage, our production department will prepare your paper for publication. This includes ensuring the following:

* All references, tables, and figures are properly cited

* All relevant supporting information is included in the manuscript submission,

* There are no issues that prevent the paper from being properly typeset

You will receive further instructions from the production team, including instructions on how to review your proof when it is ready. Please keep in mind that we are working through a large volume of accepted articles, so please give us a few days to review your paper and let you know the next and final steps.

Lastly, if your institution or institutions have a press office, please let them know about your upcoming paper now to help maximize its impact. If they'll be preparing press materials, please inform our press team within the next 48 hours. Your manuscript will remain under strict press embargo until 2 pm Eastern Time on the date of publication. For more information, please contact onepress@plos.org.

You will receive an invoice from PLOS for your publication fee after your manuscript has reached the completed accept phase. If you receive an email requesting payment before acceptance or for any other service, this may be a phishing scheme. Learn how to identify phishing emails and protect your accounts at https://explore.plos.org/phishing.

If we can help with anything else, please email us at customercare@plos.org.

Thank you for submitting your work to PLOS One and supporting open access.

Kind regards,

PLOS One Editorial Office Staff

on behalf of

Dr. Yee Gary Ang

Academic Editor

PLOS One
